# Social versus nonsocial visual cues of trustworthiness uniquely influence trust related behavior and memory

**DOI:** 10.1038/s41598-025-17094-y

**Published:** 2025-10-16

**Authors:** Jordan Schotz, Trenton Lam, Natalie C. Ebner, Nichole R. Lighthall

**Affiliations:** 1 Department of Psychology, University of Central Florida, Orlando, USA; 2 Pritzker School of Medicine, University of Chicago, Chicago, USA; 3 Department of Psychology, University of Florida, Gainesville, USA

**Keywords:** Trust, Decision making, Impression formation, Attribution theory, Social, Psychology, Human behaviour

## Abstract

Trust-related impressions and behavior are influenced by behavioral and non-behavioral (e.g., visual) cues. Similarly, nonsocial cues of trustworthiness (e.g., color cues like red or green, symbols such as warning signs or checkmarks) can influence behaviors associated with trust and distrust in memory and choice tasks. It is unclear, however, whether such social and nonsocial visual cues of trustworthiness have similar effects on behavior over iterative experiences and on post-task associative memory. To address these gaps, the Multi-Round Trust Game was used to determine effects of visual cues of trustworthiness on trust-related decision-making in social versus nonsocial economic game contexts. “Trustees” were represented by visual cues of trustworthiness (i.e., trustworthy and untrustworthy-looking faces, respectively). Social and nonsocial visual cues with comparable perceived trustworthiness yielded similar biasing effects on initial investments and investments over repeated interactions. However, social relative to nonsocial cues differentially influenced investment behavior with relatively more subtle visual cue differences (e.g., nonemotional facial characteristics versus positive/negative scenes). Additionally, social and nonsocial post-game memory was generally more distinctive, particularly for trustees who had shown untrustworthy behavior during the game. Extending *Attribution* and *Betrayal Aversion* theories, these findings suggest that while social and nonsocial visual cues can have similar effects on trust-related cognition and behavior, social relative to nonsocial cues affect behavior via more subtle visual differences, and their influence on post-interaction memory exceeds effects on trust-related behavior. These results highlight the privileged status of social information in dual-process models and support the critical role of intuitive evaluability in shaping behavior and memory across diverse trust-relevant contexts.

**Supplementary Information:**

The online version contains supplementary material available at 10.1038/s41598-025-17094-y.

## Introduction

Trust-related decisions are rarely made with complete information^1^. Instead, trust in others is typically based on limited and uncertain information^2^ that is updated as more details become available. Miscalibrated decisions of trust can have significant impacts on health and well-being. For example, overestimations of another’s trustworthiness can increase the risk of fraud and deception, while underestimations of trustworthiness can increase the risk of social isolation and loneliness^3^.

### Effects of visual cues of trustworthiness on impressions of trust

Visual cues of trustworthiness, particularly facial cues, have a potent influence on trust-related judgments and behavior, especially during impression formation^4^. For example, initial impressions of social partners are driven by emotional facial expressions^5^, static facial characteristics^6–9^, and implicit cues of interpersonal distance^10^. As a result of such visual cues, initial evaluations of trust can be positively or negatively biased^11^.

Support for the impact of visual cues of trustworthiness on perceived trustworthiness, trust behavior, and trust-related memory converges across the literature^4,12^. It remains unclear whether these effects are specific to social stimuli or extend to nonsocial entities that require trust for effective interactions (e.g., ATMs, websites that receive private information, objects, and institutions), thereby applying more broadly to visual cues of trustworthiness. Importantly, interactions with non-human agents are becoming more commonplace. In fact, past research on trust-related decision making has not directly compared the impact of visual cues of trustworthiness in social and nonsocial contexts in a manner that involves both initial impressions and repeated interactions.

Available evidence indicates that, in some situations, trust in non-human entities (*“nonsocial trustees,”* such as ATMs, robots, and computers) exceeds trust in fellow humans^13^. Additionally, akin to facial indicators of trustworthiness in social decision games, nonsocial visual cues can prompt behaviors associated with trust and distrust, both when the meaning of these cues is provided implicitly^14,15^ or explicitly^16,17^. Even subtle cues can exert a significant influence on trust-related judgments and behaviors. For example, colors are associated with concepts that differentially impact trust-related judgments and decisions, with green signaling trustworthiness and red signaling untrustworthiness in Western cultures^18–21^. These studies collectively indicate that even subtly “valenced” nonsocial visual cues (e.g., colors) can exert a pattern of effects that is similar to those of trustworthy- and untrustworthy-looking faces on trust-related impressions and associated behaviors. However, past research has also suggested that social feedback is unique and potentially more salient than nonsocial feedback^22^; though effects may be task-dependent^23^. What is unclear to date is whether nonsocial visual cues of trustworthiness have a similar impact on impressions and trust-related choices over repeated interactions, as do facial cues of trustworthiness; with repeated interactions representative of many real-life encounters/scenarios faced around trust and distrust (e.g., deciding whether to use self-checkout kiosks, public ATMs, or color-coded indicators).

In this paper, we operationalized Visual Cue Trustworthiness as the extent to which visual cues were subjectively perceived as trustworthy or untrustworthy. Specifically, “trustworthy-looking” cues were those visually perceived as signaling trustworthiness, whereas “untrustworthy-looking” cues were those visually perceived as signaling untrustworthiness. This operationalization was confirmed through independent norming data and data collected in Experiments 1 and 2.

*Attribution Theory*^24^ provides insight into differentiating social and nonsocial trust-related decision-making, suggesting that visual cues of trustworthiness may yield different effects on behavior toward social (e.g., human) than nonsocial (e.g., inanimate) entities. The theory posits that making sense of an outcome involves attributing its cause to either the “agent’s disposition or the agent’s situation”^25^. This process involves attributing agency and imagining a partner’s intentions when making sense of their betrayals^26^. The inability to attribute intentions to nonsocial entities, and thus assign blame for their actions, may also influence the interplay between visual cues and behavior. This process could lead to a less pronounced impact on trust behavior in nonsocial contexts due to differences in attribution^27^.

Furthermore, *Betrayal Aversion Theory*^28^ suggests that decision-makers exhibit greater aversion to uncertainties arising from social interactions relative to interactions with nonsocial or natural sources (e.g., lottery-based gambles, computer-generated outcomes, or environmental chance events^26,28,29^). This account implies that, in contexts involving nonsocial partners where uncertainty is perceived as less threatening, individuals may be more likely to exhibit trusting behaviors from the outset. In contrast, in interactions with social partners, individuals tend to closely scrutinize physical appearance as a method of assessing trustworthiness^30^, largely due to the deeply aversive nature of betrayal and the resulting desire to avoid it. Additionally, *Betrayal Aversion Theory* proposes that losses resulting from interactions with nonsocial compared to social entities elicit different emotional responses and, specifically, are perceived as less negative^26^.

Additional theoretical work suggests that although social and nonsocial cues can both impact behavior through affective information (*Affect Heuristic Theory*^31^), social cues are likely more impactful due to their greater intuitive accessibility and evaluability. According to the *Evaluability Hypothesis*^32^, individuals are better able to interpret and weigh cues when they have more experience or contextual grounding to assess the meaning of such cues. Given that humans are evolutionarily and experientially attuned to facial information, social cues such as facial trustworthiness are typically more interpretable and diagnostically rich than nonsocial cues. As a result, trust judgments may reflect not only the trustworthiness of the cue, but also how easily that cue can be evaluated cognitively and affectively. This interpretability advantage also aligns with *Dual-Process* theories of decision-making^33^, which propose that initial judgments under uncertainty are primarily governed by fast, intuitive processes (System 1). Because faces are readily processed by System 1 in contexts of trust-related decision making, they may exert a stronger immediate effect on trust-related decisions than nonsocial visual cues.

Collectively, these theories suggest that the effects of visual cues of trustworthiness on judgments and behavior are likely greater in social than nonsocial contexts, both for initial impression-based choices and over repeated choice-outcome experiences. Nevertheless, these theories and prior research suggest that trust overall is likely greater for nonsocial than social trustees.

### Effects of visual cues of trustworthiness on memory for social and nonsocial trustees

Moreover, an increasingly relevant consideration concerns whether visual cues of trustworthiness have a differential influence on subsequent item and associative memory for social versus nonsocial entities^34^. This consideration is growing in importance as artificial intelligence and digital interfaces replace many of the social human-to-human interactions of the past. Broadly, information relating to expectancy violations^35^ and losses^36^ is particularly memorable. Memory-based impressions are likely influenced by factors such as visual cues as well as reputational information, across social and nonsocial contexts; however, social visual cues may have a stronger impact than nonsocial visual cues due to their intrinsic memorability^37^. Indeed, in social contexts, betrayals were associated with greater memory than positive social exchanges^38^, as were untrustworthy visual cues relative to trustworthy visual cues^39^. However, findings suggesting enhanced memory for cheaters are inconsistent across studies^40,41^. Further, faces may be more salient^23^ and more easily remembered than complex non-face stimuli (e.g., scenes or objects composed of multiple elements,^42^). Taken together, these prior studies suggest that both item memory and associative memory will be greater for social than nonsocial trustees, particularly for social partners who violated social norms by betraying acts of trust, as will be tested here.

### Purpose of this study

To address gaps in the literature, this study conducted two experiments comparing trust-related behavior and memory for social and nonsocial “trustees” using a Multi-Round Trust Game. The study examined these outcomes for trustees represented by social versus nonsocial visual cues of trustworthiness, matched by perceived trustworthiness. We further manipulated trustees’ reciprocity rates to determine the impact of congruent versus incongruent cues (between visual cues and behavior) on trust-related learning and memory across social and nonsocial versions of the task.

Following prior literature, and in line with predictions by *Attribution* and *Betrayal Aversion* theories, we tested three specific hypotheses. First, based on evidence demonstrating that facial trustworthiness influences actual financial decisions in social contexts^43,44^, and from research showing that valenced nonsocial visual cues, such as color, similarly impact behavioral responses^19,45^, we predicted that greater initial investments for trustees represented by visual cues would be associated with more trust behavior, in both the social and the nonsocial Task Versions of the game (*Hypothesis 1a*). However, we expected the effects of visual cues of trustworthiness to be greater in the social than the nonsocial Task Version (*Hypothesis 1b*).

In addition, we hypothesized that trustees associated with trustworthy-looking visual cues but incongruent low reciprocity rates (e.g., “wolf in sheep’s clothing”) would receive lower investments across trials than trustees associated with untrustworthy-looking cues and congruent low reciprocity rates (*Hypothesis 2a*). Further, this Visual Cue Trustworthiness-by-Reciprocity Rate congruency interaction was expected to be greater in the social than the nonsocial Task Version (*Hypothesis 2b*).

Lastly, we predicted that visual cues of trustworthiness would bias associative memory for both social and nonsocial partners/entities; such that in both the social and the nonsocial task versions, post-game impressions that index associative memory would be more positive for trustees associated with trustworthy-looking visual cues and less positive for those associated with untrustworthy-looking visual cues (*Hypothesis 3a*). This effect, however, was expected to be greater for social than nonsocial visual cues, characterized by an interaction between Visual Cue Trustworthiness and Task Version (*Hypothesis 3b*).

## Experiment 1

### Methods

#### Participants

A power analysis was conducted using G*Power 3.1^46^ to determine the necessary sample size for a full factorial mixed-design ANOVA with one between-subjects factor (two levels) and two within-subjects factors (two levels each). The analysis was set to detect a small effect size (ƒ = 0.10), with an alpha level of 0.05 and a power (1 − β) of 0.90. Results of this power analysis indicated a total sample size of 180 (with equal numbers per between-subjects group) would be required to achieve the desired power.

A sample of 307 undergraduate participants aged 18–35 was recruited through the psychology SONA participant pool at the University of Central Florida and completed the online experimental protocol in locations of their choosing. Forty-two participants were excluded from the analysis for failing the social Task Version manipulation check, resulting in a final analysis sample of 266 participants (Table 1). Before participating in the experiment, all participants read and agreed to the informed consent form. No identifying information is published in this study. All data were anonymized prior to analysis, and no individual can be identified from the data presented. The University of Central Florida Institutional Review Board approved all aspects of the experiment, and all methods were performed in accordance with the relevant guidelines and regulations.

**Table 1:** Participant demographics.

Factor	Social	Nonsocial	Total Sample
Gender			
n	114	152	266
% Male	19.5	32.5	25.1
% Female	77.1	66.2	74.9
Age			
Mean	20.0	19.5	19.7
SD	2.6	2.9	2.3

Values reflect sample size after exclusions. Participants were given binary response options to report gender but were not required to respond.

### Procedure

The Multi-Round Trust Game was employed in this study toexamine the extent to which visual cues of trustworthiness for social and nonsocial trustees affected subsequent behavior and memory. The experiment was programmed in PsychoPy3 and administered online using Pavlovia (during the COVID-19 pandemic while University protocols prohibited in-person data collection). The study was described to participants as examining how trust is built and maintained among adults and how that affects decision making (see supplementary material). Participants completed a demographic survey through Qualtrics before being redirected to Pavlovia, where they read task instructions and performed two practice trials. Visual cues in the practice trials were cartoon computer images and thus did not include any images encountered in the experimental task. Participants then completed four blocks of the experimental task, immediately followed by post-experiment measures of perceived trustworthiness, item memory, and associative memory, as well as a manipulation check whether participants in the social task version believed their partners were real human agents. Finally, participants were redirected to Qualtrics again, where they were debriefed about the deception associated with the social manipulation of the experiment. Participants provided permission for the use of their data once the deception was disclosed. No participant denied permission.

### Stimuli

**Fig. 1:**
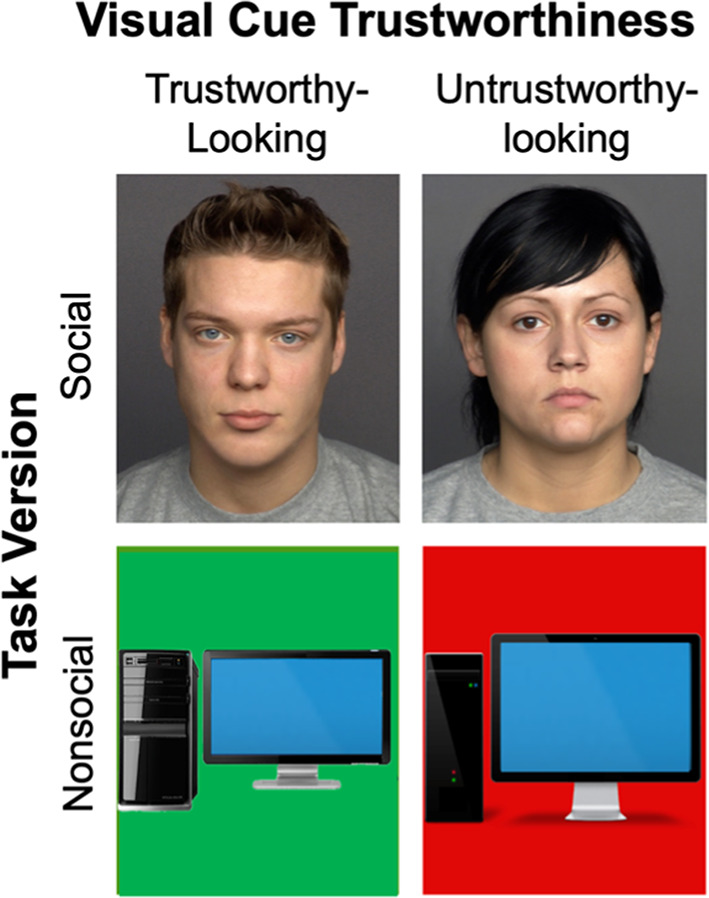
Visual cues used in Experiment 1. *Note. *The specific faces (FACES;^47^) shown here were not used in the experiment but are presented here due to reprinting permissions.

The experiment manipulated visual cues of trustworthiness for face images in the social Task Version and computer images in the nonsocial Task Version (Fig. 1). “Social partners” were represented by face images from the FACES database^47^. Independent ratings of trustworthiness were utilized^48^, in which the average rating of faces with neutral expressions, averaged between rater gender and rater age group, was utilized to select the four young-adult male faces and four young-adult female faces with the lowest and highest trustworthiness ratings for use in this study (i.e., two trustworthy-looking young men, two untrustworthy-looking young men, two trustworthy-looking young women, and two untrustworthy-looking young women). Participants were informed that the photos representing each of their game partners/trustees were selected by the partners themselves from a set of images. However, no additional details were provided regarding selection options or criteria, such as whether partners chose photos based on physical resemblance or matching gender. Specifics about the task instructions can be found in the supplementary material.

To manipulate cues of trustworthiness in the nonsocial task version, trustees were represented by images of computers on backgrounds with a solid color fill. Colors were selected based on their association with implicit biases towards trust (green) and distrust (red) behavior, respectively^16,20,49,50^. Computer image stimuli were limited to photorealistic images (not illustrative), non-salient computer wallpaper images, depictive of a desktop computer (not a laptop), and subjectively evaluated modern computer design with a front-facing screen. A solid-colored rectangle sized to match the face photographs was used as a background for the computer images (red hex code: #e10808, green hex code: #4d9a1a). Participants were not informed about how the computer images were selected. Instead, they were simply informed that the computer programs they would interact with were represented by computer avatars.

### Multi-round trust game

Participants played the Multi-Round Trust Game^51,52^ with four “partners.” Social and nonsocial Task Versions were counterbalanced between subjects. In the social task version, instructions led participants to believe that partners were human players, although, in both the social and the nonsocial Task Versions, they were not. While instructions suggested that participants could potentially have played either role (“investor” or “trustee”), all participants played the role of “investor.” Following their assignment to the role of investor, participants were informed of task details from their perspective only. Participants were not provided details about how many investors each trustee would encounter or what additional tasks trustees would be asked to do. As such, participants were not provided with specific details regarding what trustees were doing during periods when they were not actively interacting with them throughout the game.

Images of social partners were pseudo-randomly selected from a pool of eight images divided equally by gender and Visual Cue Trustworthiness (male-presenting/female-presenting, trustworthy-looking /untrustworthy-looking). Nonsocial stimuli were pseudo-randomly selected from a separate pool of eight nonsocial images and were balanced by the trustworthiness of their visual cues (trustworthy-looking/untrustworthy-looking). It was never indicated to participants that background color held any meaning or relevance to in-task outcomes; rather, any association was implicitly assumed^20^. Among the four partners encountered in the game, the probability of reciprocation was counterbalanced such that two partners reciprocated on three of the 15 trials (low reciprocity rate; 20%) and two partners reciprocated on twelve of the fifteen trials (high reciprocity rate; 80%), with the order of reciprocations pseudorandomized. In addition, each participant in the social Task Version played with one trustworthy (high reciprocity rate; 80%) and one untrustworthy (low reciprocity rate; 20%) partner of each gender (male-presenting or female-presenting) to address potential confounds from partner gender on trustworthiness perceptions.

**Fig. 2:**
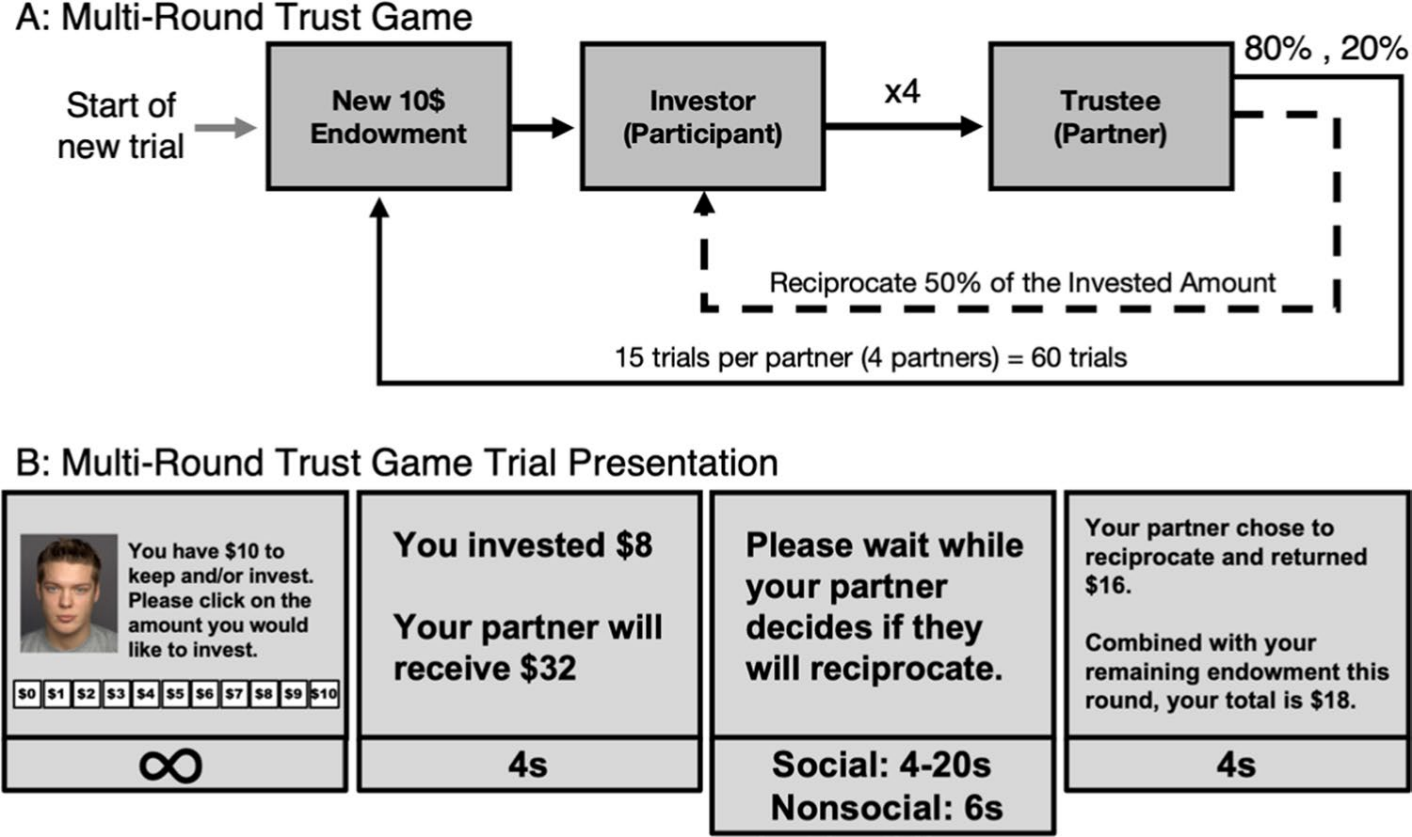
Trial Sequence of the Multi-Round Trust Game. *Note. *(Panel A) The experimental design of the Multi-Round Trust Game was administered equivalently in Experiments 1 & 2. The investor started each trial with an endowment of $10. They then chose a whole dollar amount to invest in the trustee. The invested amount appreciated (multiplied by 4) as the investor waited for the trustee to decide whether they would reciprocate on the investment. If they did, 50% of the received amount was returned to the investor. If they did not, 0% was returned to the investor. Each subsequent trial started with a new $10 endowment for a total of 15 trials per investor:trustee pair. (Panel B) The experimental design of the Multi-Round Trust Game was administered equivalently in Experiment 1 & 2. The participant made investment decisions by clicking on-screen buttons reflecting whole dollar amounts ($0–$10). Participants saw confirmation of their selected investment and the amount their partner would receive. After a waiting period, participants were presented with the received amount as well as their total earnings for that trial.

Participants waited for their game partners to reciprocate for a jittered amount of time in the social Task Version (4–20 s) to reinforce the believability of the social manipulation. Additionally, before being paired with each partner, participants waited a jittered period of 4–20 s for their next partner to “connect” to further reinforce believability of the social manipulation that the Trust Game involved other human participants that logically were not “ready” immediately, as a computer would be. Participants in the nonsocial Task Version did not wait between partners (see Fig. 2).

### Post-experiment measures

For both the social and nonsocial task versions, after completing the Multi-Round Trust Game, participants answered three questions about each of eight “partners” (four *encountered* in the game and four *unencountered* foils). The order of partner images for the post-experiment questions was pseudorandomized. In particular, participants were first asked to provide ratings of perceived trustworthiness for both encountered (visual “partner” cues they saw in the Trust Game) and unencountered partners (foil cues). Critically, post-experiment perceived trustworthiness for *unencountered* partners allowed for a manipulation check of Visual Cue Trustworthiness without confounding effects from prior experiences during the Trust Game. Post-experiment perceived trustworthiness for *encountered* partners was used as an indirect measure of associative memory for partners and their behavior during the game. We found that reciprocation rate biased trustworthiness ratings, dependent on sufficient associative memory. Perceived trustworthiness was reported on a Likert scale from 1 = ‘very untrustworthy’ to 7 = ‘very trustworthy’, represented by on-screen buttons that participants used a mouse to select.

Then, participants completed a memory assessment for encountered and unencountered partners (item memory). Participants’ item memory were collected using a 7-point Likert scale, where ‘1’ indicated ‘definitely not’ having encountered the trustee in the game and ‘7’ represented ‘definitely’ having encountered the trustee in the game. Corrected recognition scores were then calculated by subtracting average ratings for unencountered trustees from ratings for encountered trustees. For instance, if an encountered trustee was rated as ‘7’ (i.e., highly confident of having encountered) and unencountered trustees with the same visual cue type averaged ‘2’ (i.e., fairly confident of not having encountered), the corrected recognition score would be 7–2 = 5, where higher values indicated stronger item memory. This corrected recognition score ensured an intuitive interpretation of the memory performance scores.

The third and final post-experiment measure provided a second assessment of memory for partner behavior (associative memory). Specifically, for partners who received item memory ratings of 2—7 (i.e., indicating at *least some* possibility that they had been encountered in the Multi-Round Trust Game), participants were asked to estimate the portion of trials each partner chose to send money back (reciprocate). Estimates were made using a slider with percentages ranging from 0 to 100% (note that during the experiment, the true reciprocation rates of 20% and 80% were never stated numerically to participants).

### Believability ratings

In the social task version, participants then provided ratings indicating their belief in the social manipulation^34^. This procedure was conducted to determine the degree to which participants believed their partners were human agents. As in FeldmanHall et al., participants who reported “7” on a Likert scale (7 = ‘definitely did not believe’, 1 = ‘definitely believed’) were excluded from subsequent analyses (Experiment 1 = 25.8%; Experiment 2 = 9.7%). Participants in the nonsocial task version were not presented with this question or any alternative form.

## Results

### Trust-related behavior (Hypothesis 1)

**Fig. 3:**
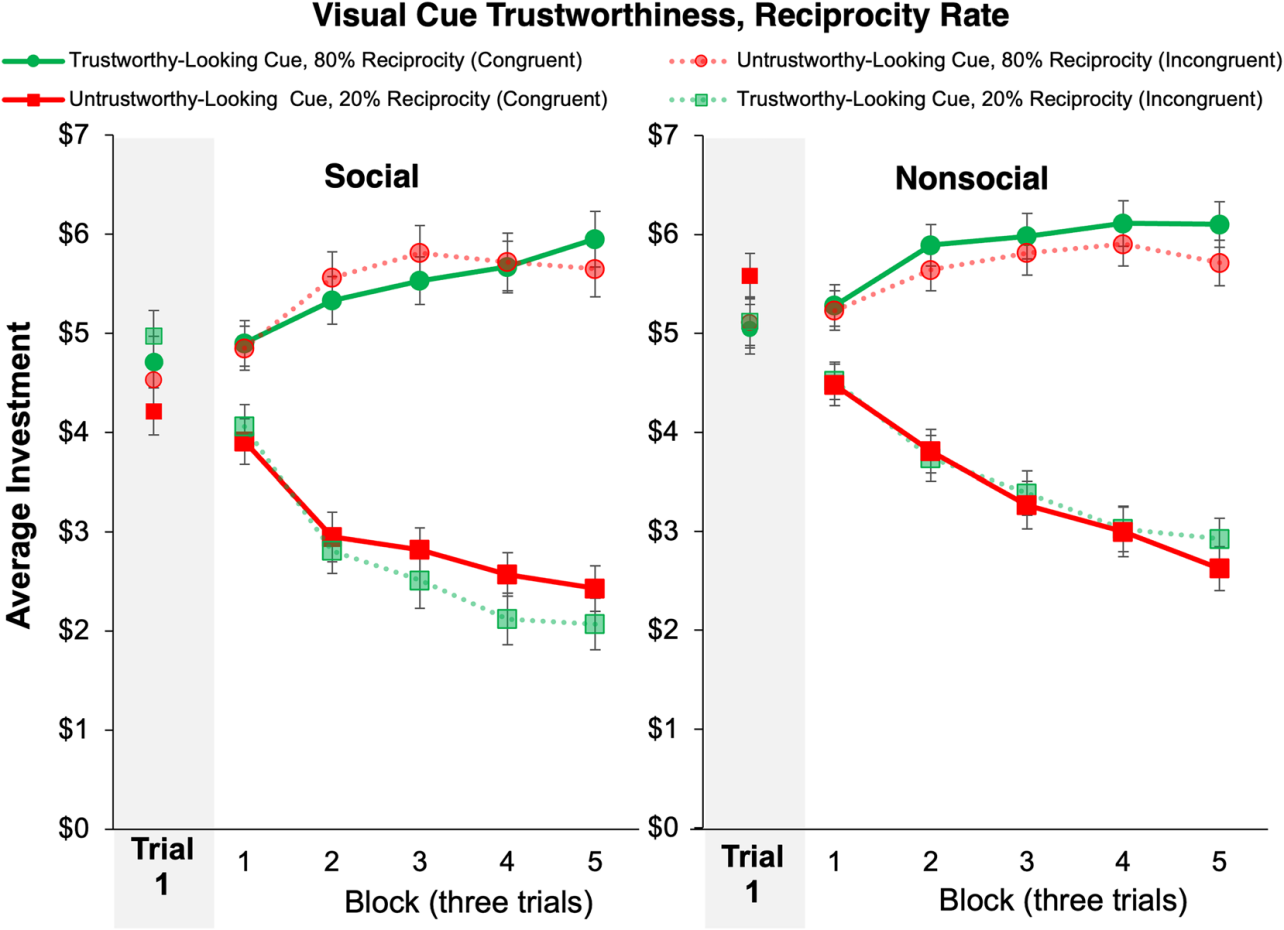
Learning across blocks of 3 trials in the Multi-round Trust Game. *Note*. “Congruent” refers to partners whose Visual Cue Trustworthiness matched their behavioral trustworthiness: i.e., trustworthy-looking cues paired with high reciprocity rates (80%) or untrustworthy-looking cues paired with low reciprocity rates (20%). “Incongruent” refers to mismatched pairings (e.g., a trustworthy-looking cue paired with low reciprocity). Error bars represent ± 1 SEM.

To test *Hypothesis 1a* that visual cues associated with trustworthiness would lead to higher initial investments in both the social and the nonsocial task versions, initial investments (prior to any exchange with the partner) were analyzed with a full-factorial 2 (Task Version: Social, nonsocial) × 2 (Visual Cue Trustworthiness: Trustworthy-looking, untrustworthy-looking) mixed model ANOVA. The main effect of Visual Cue Trustworthiness was nonsignificant (*F*(1,270) = 0.54, *p* = 0.46, *η*^2^ < 0.001), thus not supporting *Hypothesis 1a*. However, the interaction between Visual Cue Trustworthiness and Task Version was significant (*F*(1,270) = 7.36, *p* = 0.01, *η*^2^ = 0.01). As shown in the shaded region in Fig. 3, this interaction indicated that initial investments were higher for trustworthy-looking than untrustworthy-looking visual cues in the social, but not in the nonsocial task, version, supporting *Hypothesis 1b*. Results also revealed a main effect of Task Version, such that initial investments were higher in the nonsocial (*M* = 5.21, *SEM* = 0.17) than in the social (*M* = 4.58, *SEM* = 0.19) (*F*(1,270) = 6.16, *p* = 0.01, *η*^2^ = 0.02) Task Version.

### Trust-related learning (Hypothesis 2)

Next, to test *Hypothesis 2a* that trustees associated with trustworthy-looking visual cues but incongruent (low) reciprocity rates (e.g., “wolf in sheep’s clothing”) would receive lower investments across trials than trustees associated with untrustworthy-looking cues and congruent (low) reciprocity rates and that the effect would be stronger in the social Task Version (*Hypothesis 2b*), we examined average investment across time using a full-factorial 2 (Task Version: Social, nonsocial) × 2 (Reciprocity Rate: High, low) × 2 (Visual Cue Trustworthiness: Trustworthy-looking, untrustworthy-looking)×5 (Block: Block 1, Block 2, Block 3, Block 4, Block 5) mixed factor ANOVA where blocks were comprised of three trials of the total fifteen for each partner, resulting in five blocks of interactions for each investor-trustee pair following Bailey et al.^6^. Results indicated significantly greater investment across trials for partners with high (*M* = 5.63, *SEM* = 0.12) versus low (*M* = 3.16, *SEM* = 0.12) (*F*(1,270) = 438.04, *p* < 0.001, *η*^2^ = 0.16) reciprocation rates, indicating that participants were responsive to varying rates of reciprocation in the direction expected. However, no interaction between Reciprocity Rate and Visual Cue of Trustworthiness was found (*p* = 0.27, *η*^2^ < 0.001), thus not supporting *Hypothesis 2a*. A follow-up exploratory 2 (Task Version: Social, nonsocial) × 2 (Cue Congruence: Congruent, incongruent) × 5 (Block: 1–5), where Cue Congruence was coded by whether Visual Cue Trustworthiness was congruent with the Reciprocation Rate. This analysis, described in greater depth in the supplementary material, found no significant effect of Cue Congruence or an interaction between Task Version and Cue Congruence, further demonstrating a lack of support for *Hypothesis 2a*. The interaction of Reciprocity Rate × Visual Cue Trustworthiness × Task Version that tested *Hypothesis 2b* was nonsignificant (*p* = 0.96, *η*^2^ < 0.001) in this analysis; and no main effect of Visual Cue Trustworthiness was observed (*F*(1,270) = 0.001, *p* = 0.97, *η*^2^ < 0.001).

Interestingly, and consistent with our findings for initial investments, average investments were higher in the nonsocial (*M* = 4.63, *SEM* = 0.14) than the social (*M* = 4.16, *SEM* = 0.16) (*F*(1,270) = 5.31, *p* = 0.02, *η*^2^ = 0.01) Task Version. Further, as shown in Fig. 3, there was a main effect of block (*F*(1, 270) = 9.44, *p* < 0.001, *η*^2^ = 0.003), with investments declining over blocks (Block 1: *M* = 4.68, *SEM* = 0.12; Block 2: *M* = 4.47, *SEM* = 0.12; Block 3: *M* = 4.39, *SEM* = 0.12; Block 4: *M* = 4.26, *SEM* = 0.12; Block 5: *M* = 4.18, *SEM* = 0.12); and a significant Block × Reciprocity Rate interaction (*F*(1,270) = 52.92, *p* < 0.001, *η*^2^ = 0.02) indicating enhanced learning when interacting with partners with low compared to high reciprocity rates. No other effects in this analysis reached significance (*p* > 0.07).

### Post-experiment memory ratings (Hypothesis 3)

After the Multi-Round Trust Game, participants immediately rated their memory for the four encountered and four additional unencountered (foil) “partners.” A 2 (Task Version: Social, nonsocial) × 2 (Reciprocity Rate: High, low) × 2 (Visual Cue Trustworthiness: Trustworthy-looking, untrustworthy-looking) ANOVA was conducted on corrected recognition scores to assess item memory for trustees. Scores could range from − 6 to 6, where “6” indicated certainty that encountered partners were encountered and that unencountered partners were not encountered, “− 6” indicated the opposite (i.e., certainty in both false alarms and omissions), and “0” indicated chance performance. The intercept for the model was 3.60 (*SEM* = 0.13, *p* < 0.001), and post-hoc one-sample t-tests showed that item memory for trustees was above chance across Task Versions (*p* < 0.001). Additionally, results showed a significant effect of Task Version (*F*(1,270) = 78.96, *p* < 0.001, *η*^2^ = 0.15), indicating better memory for social (*M* = 4.72, *SEM* = 0.19) than nonsocial (*M* = 2.48, *SEM* = 0.17) partners. A main effect was found for Reciprocity Rate such that corrected recognition was greater for partners with high (*M* = 3.73, *SEM* = 0.14) than low (*M* = 3.48, *SEM* = 0.14) (*F*(1,270) = 5.03, *p* = 0.03, *η*^2^ = 0.002) reciprocity rates. No other main effects or interactions reached significance (*ps* > 0.20).

Perceived trustworthiness of encountered partners was used as a measure of associative memory for trustees and interaction outcomes. A 2 (Task Version: Social, nonsocial) × 2 (Reciprocity Rate: High, low) × 2 (Visual Cue Trustworthiness: Trustworthy-looking, untrustworthy-looking) ANOVA was conducted to test *Hypothesis 3* that trustworthy-looking visual cues bias associative memory positively while untrustworthy-looking visual cues bias associative memory negatively. Consistent with *Hypothesis 3a*, participants perceived encountered partners with trustworthy-looking visual cues as more trustworthy (*M* = 4.01, *SEM* = 0.07) than those with untrustworthy-looking visual cues (*M* = 3.66, *SEM* = 0.08) (*F*(1,270) = 13.24, *p* < 0.001, *η*^2^ = 0.01), across reciprocation rates and Task Versions. Notably, however, the effects of visual cues on perceived trustworthiness for encountered partners did not differ across the social and nonsocial Task Versions (nonsignificant Visual Cue Trustworthiness × Task Version interaction; *p* = 0.70), thus not supporting *Hypothesis 3b*.

**Fig. 4:**
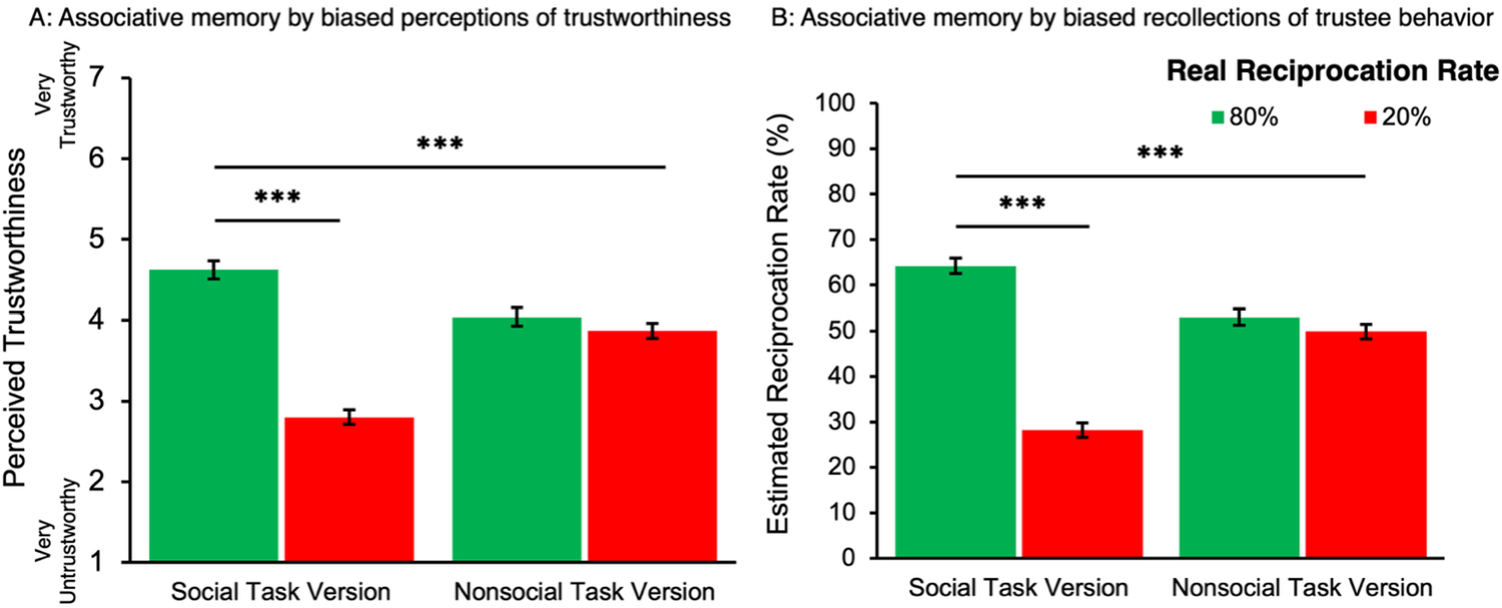
Average Ratings of Perceived Trustworthiness by Social Task Version and Reciprocity Rate. Panel A: Average subjective ratings of perceived trustworthiness for partners, reflecting associative memory for partner behavior. Participants showed greater sensitivity to reciprocity history in the social Task Version than in the nonsocial Task Version—i.e., they rated high-reciprocating social partner as more trustworthy than low-reciprocity social partners, indicating stronger associative memory in the condition. Panel B: Estimated reciprocation rates by Task Version and Reciprocity Rate. A significant Task Version×Reciprocity Rate interaction indicated greater associative memory for partner behavior in the social Task Version compared to the nonsocial Task Version. *Note*. Error bars represent ± 1 SEM. ***P < .001.

Additionally, a robust effect of Reciprocity Rate was found (*F*(1,270) = 109.44, *p* < 0.001, *η*^2^ = 0.08), with higher perceived trustworthiness for partners with high (*M* = 4.33, *SEM* = 0.08) than low (*M* = 3.34, *SEM* = 0.07) reciprocity rates, indicating that participants did track associations between partners and their reciprocity rates. We also found that encountered nonsocial partners were perceived to be more trustworthy (*M* = 3.96, *SEM* = 0.08) than encountered social partners (*M* = 3.71, *SEM* = 0.09), evidenced by a significant main effect for Task Version (*F*(1,270) = 4.55, *p* < 0.001, *η*^2^ = 0.01) on perceived trustworthiness for encountered partners. Task Version furthermore interacted with Reciprocity Rate (*F*(1,270) = 76.13, *p* < 0.001, *η*^2^ = 0.06; Fig. 4a), with pairwise comparisons showing that associative memory (i.e., partner identity with partner “behavior”) was greater for social (*p* < 0.001) than nonsocial (*p* = 0.19) partners. No other effects from this analysis reached significance (*p* > 0.37).

Additionally, a 2 (Task Version: Social, nonsocial) × 2 (Reciprocity Rate: High, low) × 2 (Visual Cue Trustworthiness: Trustworthy-looking, untrustworthy-looking) ANOVA was conducted on reciprocity rates estimated by participants in the post-task phase. Recall that the real reciprocation rates for high and low reciprocity conditions were 80% and 20%, respectively. Across Task Versions and real reciprocity rates, participants estimated higher reciprocity rates for partners with trustworthy-looking (*M* = 51.10%, *SEM* = 1.18%) than untrustworthy-looking (*M* = 46.62%, *SEM* = 1.17%) (*F*(1,193) = 10.43, *p* = 0.001, *η*^2^ = 0.01) visual cues. These findings for the main effect of Visual Cue Trustworthiness supported *Hypothesis 3a*. However, the effect of Visual Cue Trustworthiness did not interact with Task Version (*p* = 0.24, *η*^2^ = 0.001), thus not supporting *Hypothesis 3b*. Our analysis also showed a significant interaction between Reciprocity Rate and Task Version (*F*(1,193) = 122.14, *p* < 0.001, *η*^2^ = 0.11; Fig. 4b). Post-hoc paired samples t-tests within each task version yielded significant differences in perceived trustworthiness by Reciprocity Rate for the social (*t*(117) = − 11.95, *p* < 0.001) but not the nonsocial (*t*(151) = − 1.39, *p* = 0.17) task version. This result suggests that associative memory (i.e., partner identity with partner “behavior”) was greater for social than nonsocial partners. Lastly, a significant effect for Reciprocity Rate was found in that partners with high reciprocation rates were estimated to have reciprocated more often (*M* = 58.61%, *SEM* = 1.17%) than partners with low reciprocation rate (*M* = 39.11%, *SD* = 1.24%) (*F*(1,193) = 171.05, *p* < 0.001, *η*^2^ = 0.15), replicating trends found for perceived trustworthiness of encountered partners. No other effects reached significance in this analysis (*ps* > 0.37).

### Manipulation check

**Fig. 5:**
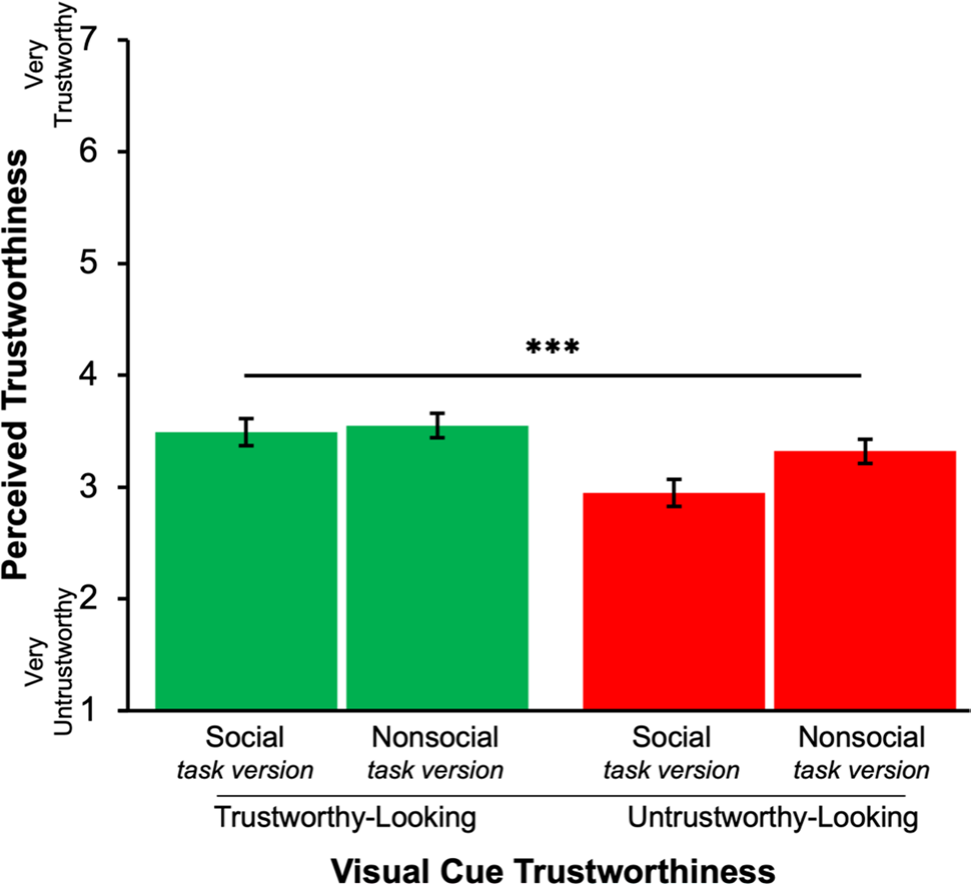
Perceived Trustworthiness Rating for Unencountered Partners. *Note*. Error bars represent ± 1 SEM. Visual cues in the trustworthy-looking condition were rated significantly higher in perceived trustworthiness than those in the untrustworthy-looking condition across both task vesion (*P* < .001) The significance bar reflects this main effect of Visual Cue Trustworthiness, confirming the success of the manipulation Scores reflect rating of unencountered partners (foils) collected post-task as a manipulation check for the effect of Visual Cue Trustworthiness. *** *P* < .001.

To confirm visual cue manipulation effects on perceptions of trustworthiness, we conducted a 2 (Task Version: Social, nonsocial) × 2 (Visual Cue Trustworthiness: Trustworthy-looking, untrustworthy-looking) full-factorial repeated-measures ANOVA on perceived trustworthiness for unencountered partners (foil images). As depicted in Fig. 5, the expected effect of Visual Cue Trustworthiness was found (*F*(1,268) = 22.84, *p* < 0.001), such that participants rated partners associated with trustworthy-looking cues as more trustworthy (*M* = 3.52, *SEM* = 0.08) than those associated with untrustworthy-looking cues (*M* = 3.13, *SEM* = 0.08). Perceived trustworthiness did not differ by Task Version (*p* = 0.13). The interaction between Visual Cue Trustworthiness and Task Version was marginally significant (*F*(1,268) = 3.62, *p* = 0.06). Pairwise comparisons for each Task Version yielded a significant difference in perceived trustworthiness by Visual Cue Trustworthiness in both the nonsocial (*F*(1,268) = 4.74, *p* = 0.03) and the social (*F*(1,268) = 19.83, *p* < 0.001) task versions, wherein both social and nonsocial partners associated with trustworthy-looking visual cues were perceived as more trustworthy than partners associated with untrustworthy-looking cues.

Together, these results confirmed that visual cues of trustworthiness affected impressions of trustworthiness in both the social and the nonsocial task versions as expected, affirming the manipulation check.

## Discussion

Experiment 1 examined effects of visual cues associated with trust versus distrust on behavior and memory, revealing distinct patterns for social versus nonsocial contexts. Supporting *Hypothesis 1b*, we found that in *social but not nonsocial* contexts initial trust behavior was greater when interacting with partners associated with trustworthy-looking compared to untrustworthy-looking visual cues. This finding aligns with previous research on social decision making, which highlights the role of visual information in perceptions of trustworthiness. Specifically, partners represented by trustworthy-looking visual cues were perceived as more trustworthy than those associated with untrustworthy-looking visual cues^5,6^. We did, however, not find the predicted significant interaction of visual cues and trust behavior across trials, thus failing to support the expected notion of “wolf in sheep’s clothing” (*Hypothesis 2a*). Finally, in support of *Hypothesis 3b*, despite initial effects of visual cues on trust behavior, we observed a weaker association of visual and behavioral information in nonsocial than social interactions. However, for associative memory, there was no interaction between Visual Cue Trustworthiness and Reciprocity Rate. That is, the idea that trustees demonstrating untrustworthy behavior paired with trustworthy-looking visual cues would be better remembered was not supported in our data. Instead, only social visual cues had a consistent memory advantage, perhaps due to their inherent value, and in line with Shore and Heerey ^53^.

It is important to note that in Experiment 1, visual cues for social partners were conveyed through facial features (foreground), whereas visual cues for nonsocial partners were represented by background colors. While past literature suggests foreground cues typically exert stronger influence on cognition and behavior, background information can also meaningfully influence decision-making processes, particularly when foreground cues are ambiguous or subtle^54,55^. We manipulated Visual Cue Trustworthiness of nonsocial “cues” in Experiment 1 through the background of these cues. In Experiment 2, in contrast, we manipulated Visual Cue Trustworthiness of nonsocial cues through the foreground, as done for social cues. Experiment 2, therefore, directly addresses this difference in location (foreground versus background). In particular, we implemented new nonsocial visual cues in Experiment 2 that enabled a clearer comparison with the social cues. As such, Experiment 2 addressed this possible limitation by featuring valenced information in the “face” of the computer, making it more analogous to the faces used for the social cues.

Further, a possible limitation of Experiment 1 was that past histories of betrayal trauma were not considered, while literature suggests it affects future trust behavior^56,57^. Experiment 2 explicitly considered this factor by employing the Brief Betrayal Trauma Survey (BBTS) to assess betrayal trauma and its effects on how visual cues influence trust impressions, behavior, and memory. Finally, whereas Experiment 1 employed subtle (implicit) nonsocial visual cues, similar to the subtle differences in static facial characteristics, Experiment 2 tested equivalent hypotheses with more robust manipulations of visual cue trustworthiness for nonsocial partners by matching perceived trustworthiness of nonsocial visual cues to their social counterparts more equivalently and featuring that content in the foreground versus the background (Experiment 1).

## Experiment 2

### Methods

#### Participants

A sample of 206 undergraduate participants was collected using the SONA participant pool at the University of Central Florida (see Table 2). Participants were awarded a standard amount of course credit for completing the experiment, independent of their task performance, but in contrast to Experiment 1, participants also received a monetary bonus for their task performance. In particular, participants were told that the “more money you make in the game, the more money you make in real life on your Amazon Gift Card, up to $4”. Participants were also informed that the payout would be 20% of a randomly selected trial. However, participants all received the same reimbursement at the end of the study and were debriefed on the purpose of this deception, which was to induce incentive-compatible behavior. Participants were not informed about the formula by which their partner’s compensation would be determined. Participants read and agreed to the informed consent before the experiment. No identifying information is published in this study. All data were anonymized prior to analysis, and no individual can be identified from the data presented. The University of Central Florida Institutional Review Board approved all aspects of the experiment, and all methods were performed in accordance with the relevant guidelines and regulations.

**Table 2:** Experiment 2 demographic data.

Factor	Social	Nonsocial	Total sample
Gender*			
n			206
% Male	41.0	47.6	44.4
% Female	59.0	52.4	55.6
Age			
n			206
Mean	19.7	19.2	19.9
SD	2.4	1.8	2.4

Chi square test of independence showed that gender and task version were independent (*p* > 0.05).

Institutional Review Board approved all aspects of the experiment, and all methods were performed in accordance with the relevant guidelines and regulations..

### Procedure

Study procedures were maintained from Experiment 1, with a few key exceptions relating to Experiment 2’s in-person context, social manipulation cover story, the addition of a monetary bonus, and the addition of an individual difference measure of past betrayal trauma.

With respect to the in-person context and social cover story, a lead experimenter greeted and instructed participants using a similar verbal script in the social and nonsocial task versions (See supplemental material for full script). However, in the social task version, this script mentioned “other participants” who were ostensibly being set up in other rooms in the building. In both task versions, a sign on the door labeled the room as the location for ‘Investor 1’ (the participant’s role). Participants in the social task version were exposed to a modified version of the sign that additionally listed room numbers assigned to their partners in the game (see supplemental material for images of signs).

**Fig. 6:**
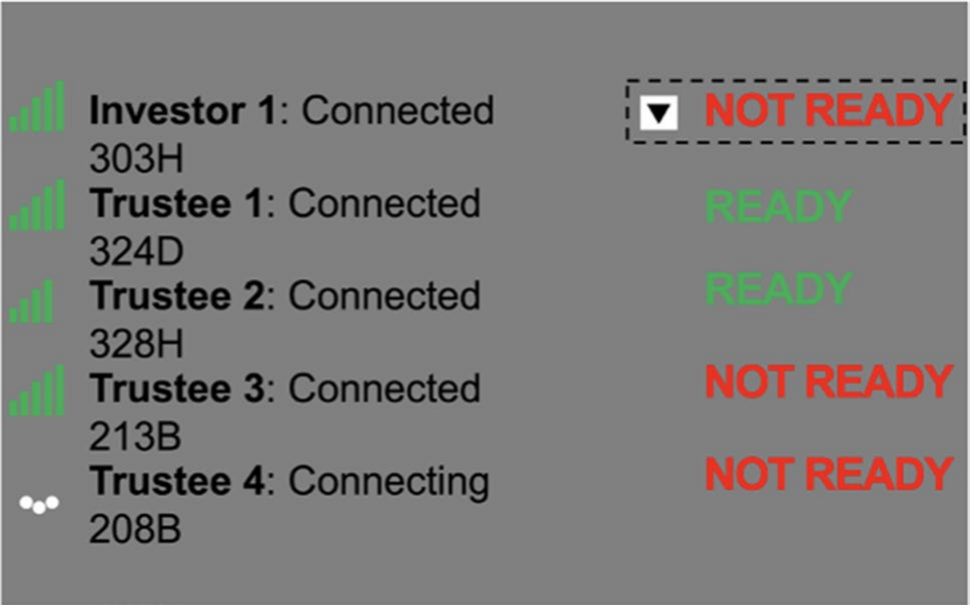
Fake social partner status monitor. *Note. *This image represents what is displayed on the experimenter’s screen; positioned 4 feet to the right of the participant’s screen. No measures were taken to ensure the degree to which the “connection” status monitor was processed or understood by the participant, though the script utilized by experimenters consistently referenced the monitor and its contents (see supplemental information).

For both task versions, upon entering the study room, a second experimenter was present along with the participant’s testing computer and a second monitor that was clearly visible to the participant while seated at their testing station. Thereafter, in the social task version, while introducing participants to the study, the lead experimenter observed a fake display on the monitor of the second computer. This displayed a slideshow, shown in Fig. 6, that was meant to track connections with the participant’s four partners in the game, and their status as “ready” to begin the experiment. This slideshow was started just prior to social-task-version participants entering their testing room. Initially, the fake display showed that one partner was ready to begin. As social-task-version participants completed informed consent, the slideshow continued to play, and two additional partner statuses advanced to “ready.” While waiting for the last partner’s status to change to ready, the two experimenters discussed whether the experimenters in the room of the last partner needed help or the possibility of the last partner being absent. The lead experimenter then asked the second experimenter to go to the room number listed for the final partner, who was not yet ready. The second experimenter then left the room, and after two minutes, the final partner’s status changed on the monitor to “ready” via a mouse click initiated by the lead experimenter, using an additional Bluetooth-connected mouse that was not visible (or audible) to the participant. Thereafter, the lead experimenter informed the participant that all partners were ready, and they could begin the experiment. The second experimenter returned to the room while participants reviewed task instructions (2.5 min after leaving; timed using a cellphone stopwatch app).

In the nonsocial task version, the second computer monitor was present but was switched off. Additionally, the second experimenter left the room at the equivalent point in time during the pre-task procedures (i.e., during the consent period) and returned 2.5 min after leaving.

With respect to the monetary compensation, the study debriefing declared the deception associated with the structure of the monetary compensation and disclosed that all participants received the maximum bonus ($4).

### Stimuli

**Fig. 7:**
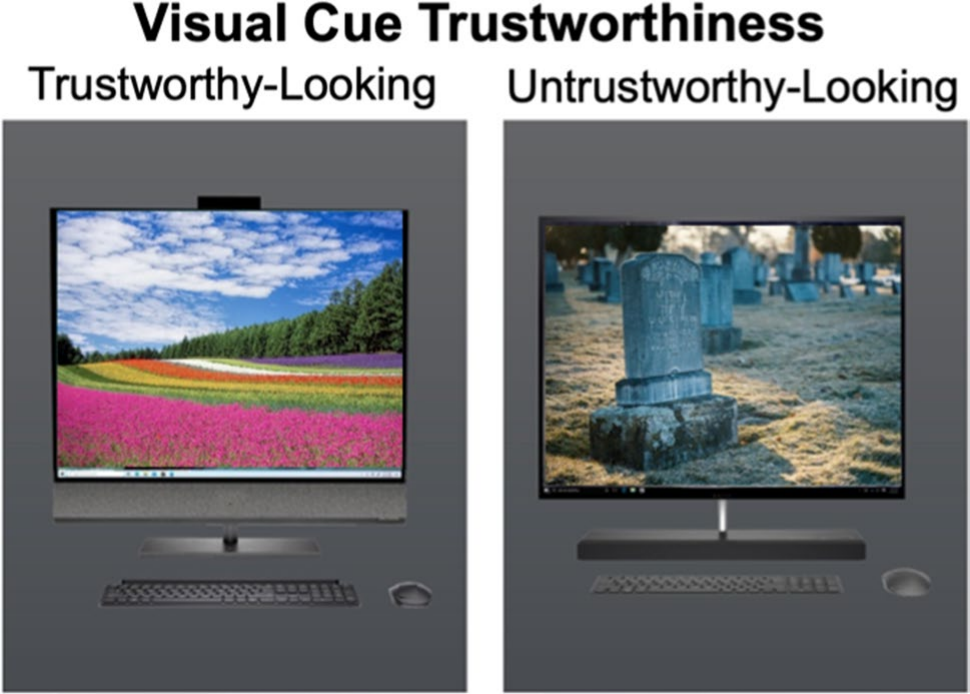
Nonsocial visual cues used in Experiment 2. *Note. *Wallpaper images are not exact reflections of the images used in the experiment. Similar images to the IAPS images employed are depicted here to reflect the stimuli used.

Stimuli utilized in the Multi-Round Trust Game followed the same structure as in Experiment 1, with changes made only to the nonsocial visual cues, as shown in Fig. 7. Nonsocial partners in Experiment 2 were depicted by images of computers with emotionally salient wallpaper images, overlayed on a gray background similar to the background for social stimuli. Computer images framing the IAPS images were selected to include only photorealistic (not illustrations) front-facing desktop computers (not laptops) of a subjectively evaluated modern design. Wallpaper images were obtained from the IAPS photo database^58^, where the four most positively rated nonsocial images and the four most negatively rated nonsocial images were used. Nonsocial images were characterized as those that contain no human beings (whole or partial, foreground or background). Pilot testing conducted on the selected set of nonsocial images showed similar effects of cue trustworthiness on perceived trustworthiness ratings for nonsocial as social visual cues.

### Multi-round trust game

The design of the Multi-Round Trust Game was identical to Experiment 1, with the exception of the images used as visual cues in the nonsocial Task Version.

### Post-experiment measures

Identical post-experiment questions as in Experiment 1 were employed in Experiment 2, including memory questions. In addition, the Brief Betrayal Trauma Survey (BBTS) was administered because literature suggests a history of betrayal trauma affects future trust behavior^56,57^. The BBTS asked participants to indicate the number of times they experienced a list of 12 prompts of various traumas, ranging from natural disasters to emotional or psychological mistreatment by someone close to them^59^. Response options were as follows: “Never,” “One or more,” or “More than that.” The prompts can be classified as either “high” or “low” betrayal traumas. For instance, experiencing an attack by an unknown perpetrator is considered a low betrayal trauma, while an attack from someone close is considered a high betrayal trauma. Thus, participants can be classified based on the number of each type of betrayal trauma experienced or based on whether a certain level of prompt was experienced at all^60^. Participants in Experiment 2 were placed in a “No Betrayal Trauma” group if they answered “Never” for all three prompts characterizing high betrayal trauma (relating to violence, sexual abuse, or emotional maltreatment by a close person). If participants answered “One or more” or “More than that” for any of these three prompts, they were classified into the “Betrayal Trauma” group. Arguably, abuse by a stranger may affect behavior in the Trust Game as participants were asked to invest in strangers. However, the current study assumed that interpersonal betrayals and trust violations done by a close person would elicit significant differences in trust behavior. For this reason, it was not appropriate to include abuse or violence by a stranger for the “Betrayal Trauma” group.

### Believability ratings

As in Experiment 1, participants were excluded from the social task version if they indicated they did not believe the social task version cover story (i.e., responded “didn’t believe at all” to the believability question). Ten participants were excluded from the following analyses on this basis, comprising 9.7% of participants in the social task version. This rate of exclusion is lower than that in Experiment 1, where 25.8% of participants in the social task version were excluded for not believing the social manipulation.

## Results

### Trust-related behavior (Hypothesis 1)

**Fig. 8:**
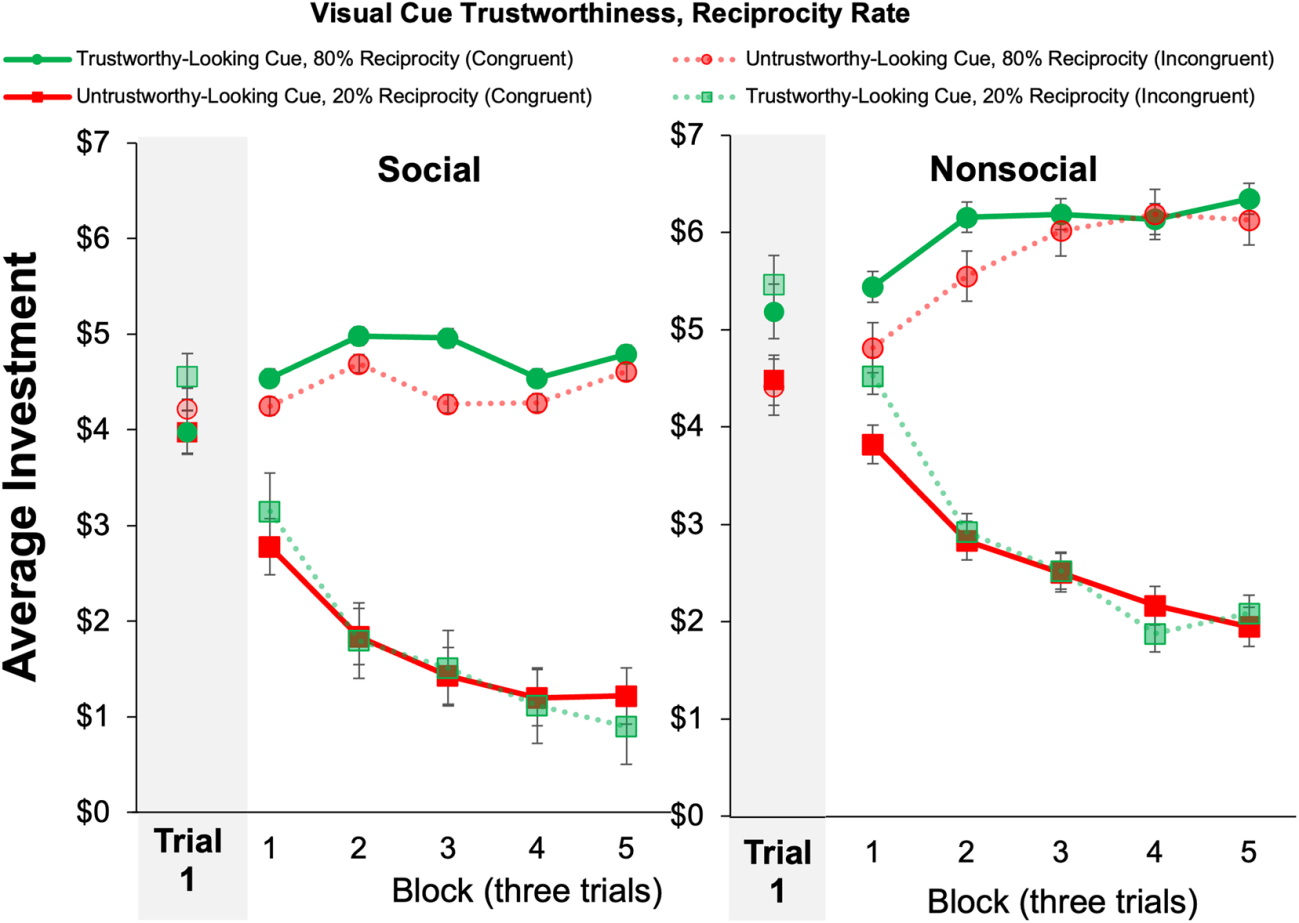
Learning across blocks of 3 trials in the Multi-Round Trust Game. *Note.* “Congruent” refers to partners whose Visual Cue Trustworthiness matched their behavioral trustworthiness: i.e., trustworthy-looking cues paired with high reciprocity rates (80%) or untrustworthy-looking cues paired with low reciprocity rates (20%). “Incongruent” refers to mismatched pairings (e.g., a trustworthy-looking cue paired with low reciprocity). Error bars represent ± SEM.

To assess trust-related behavior, a 2 (Task Version: Social, nonsocial) × 2 (Visual Cue Trustworthiness: Trustworthy-looking, untrustworthy-looking) mixed measures ANOVA was conducted, controlling for betrayal trauma. Again, the first investment made with each partner during the Trust Game prior to any interaction outcomes was used as a behavioral measure of trust based solely on visual cues. Results revealed a main effect of Visual Cue Trustworthiness on first investment (*F*(1,193) = 8.33, *p* = 0.004, *η*^2^ = 0.01). Consistent with findings in Experiment 1, trustworthy-looking visual cues were associated with higher initial investments, supporting *Hypothesis 1a*, which predicted that initial investments would be greater for partners represented by trustworthy-looking visual cues (shaded region in Fig. 8). We also observed a significant Task Version by Visual Cue Trustworthiness interaction (*F*(1,193) = 5.18, *p* = 0.02, *η*^2^ = 0.01) qualifying this main effect. Examination of means indicated that the effect of Visual Cue Trustworthiness on initial investments was present in the nonsocial but not the social Task Version. A post-hoc paired samples t-test was conducted by Visual Cue Trustworthiness on initial investments separately for social and nonsocial Task Versions. T-test results showed a significant effect of Visual Cue Trustworthiness for nonsocial (trustworthy-looking: *M* = 5.33, *SEM* = 0.22, untrustworthy-looking: *M* = 4.34, *SEM* = 0.19)(*t*(102) = 3.44, *p* < 0.001) but not social partners (trustworthy-looking: *M* = 4.24, *SEM* = 0.23, untrustworthy-looking: *M* = 4.05, *SEM* = 0.20) (*t*(92) = 1.03, *p* = 0.31), opposite to the prediction in *Hypothesis 1b*. This pattern of findings contrasts with Experiment 1, where the opposite effect was found. Further, and in alignment with results in Experiment 1, we observed a significant main effect of Task Version (*F*(1,193) = 8.58, *p* = 0.004, *η*^2^ = 0.03), where participants in the nonsocial Task Version (*M* = 4.88, *SEM* = 0.17) made higher initial investments than participants in the social Task Version (*M* = 4.14, *SEM* = 0.18).

### Trust-related learning (Hypothesis 2)

To test *Hypothesis 2a* that trustees associated with trustworthy-looking visual cues but incongruent low reciprocity rates (e.g., “wolf in sheep’s clothing”) would receive lower investments across trials than trustees associated with untrustworthy-looking cues and congruent high and low reciprocity rates and that the effect would be stronger in the social Task Version (*Hypothesis 2b*), a full factorial 2 (Task Version: Social, nonsocial) × 2 (Reciprocity Rate: High, low) × 2 (Visual Cue Trustworthiness: Trustworthy-looking, untrustworthy-looking) × 5 (Block: Block 1, Block 2, Block 3, Block 4, Block 5) mixed factor ANOVA was conducted on average investment, again with betrayal trauma added as a control factor.

The main effect of Reciprocity Rate was significant (*F*(1,193) = 264.50, *p* < 0.001, *η*^2^ = 0.14), where partners with high reciprocity rates received higher average investments (*M* = 5.26, *SEM* = 0.12) than partners with low reciprocity rates (*M* = 2.22, *SEM* = 0.09). This effect is qualified by a marginal Visual Cue Trustworthiness by Reciprocity Rate interaction (*F*(1,193) = 3.46, *p* = 0.07, *η*^2^ < 0.001), described in greater depth and shown in Figure S1 in the supplemental information. This marginal effect finds the effect of Visual Cue Trustworthiness only to be present in the high reciprocity rate condition. An exploratory 2 (Task Version: Social, nonsocial) × 2 (Cue Congruence: Congruent, incongruent) × 5 (Block: 1–5), where Cue Congruence was coded by whether Visual Cue Trustworthiness was congruent with the Reciprocation Rate. This analysis, described in greater depth in the supplementary material, found a significant effect of Cue Congruence *F*(1,193) = 9.31, *p* = 0.003) contrary to the findings of Experiment 1 and in support of *Hypothesis 2a*. The interaction of Reciprocity Rate × Visual Cue Trustworthiness × Task Version that tested *Hypothesis 2b* was nonsignificant (*p* = 0.65, *η*^2^ < 0.001).

Additionally, the main effect of Task Version was significant (*F*(1,193) = 46.54, *p* < 0.001, *η*^2^ = 0.05), such that participants who played with nonsocial partners invested significantly more (*M* = 4.33, *SEM* = 0.12) than participants who played with social partners (*M* = 3.14, *SEM* = 0.13). Further, the main effect of Visual Cue Trustworthiness was significant (*F*(1,193) = 9.31, *p* = 0.003, *η*^2^ = 0.003), which was not modulated by Task Version (*F*(1,193) = 0.09, *p* = 0.77, *η*^2^ < 0.001), but indicated that average investments were greater in partners associated with trustworthy-looking (*M* = 3.84, *SEM* = 0.10) than those associated with untrustworthy-looking (*M* = 3.64, *SEM* = 0.10) visual cues. Block-wise analyses of learning found a significant main effect of Block (*F*(1,193) = 11.33, *p* < 0.001, *η*^2^ = 0.003), where average investments decreased over time (Block 1: *M* = 4.17, *SEM* = 0.11; Block 2: *M* = 3.86, *SEM* = 0.10; Block 3: *M* = 3.69, *SEM* = 0.10; Block 4: *M* = 3.45, *SEM* = 0.10; Block 5: *M* = 3.51, *SEM* = 0.11). No other effects in this analysis reached significance (*p* > 0.15).

### Post-experiment memory ratings (Hypothesis 3)

A 7-point Likert scale was used to assess participants’ memory of playing with each partner (item memory), where “7” represented that they encountered the partner and “1” indicated that they did not encounter the partner. A 2 (Task Version: Social, nonsocial) × 2 (Reciprocity Rate: High Reciprocity Rate, low Reciprocity Rate) × 2 (Visual Cue Trustworthiness: Trustworthy-looking, untrustworthy-looking) ANOVA on corrected recognition scores with betrayal trauma added as a control factor show no significant effect of Task Version (*p* = 0.48, *η*^2^ > 0.001), indicating that social partners were recognized at a similar rate to nonsocial partners. However, a Reciprocity Rate by Task Version interaction was found (*F* (1,193) = 5.22, p = 0.02, *η*^2^ = 0.003), such that item memory was greater for participants in the nonsocial Task Version whose partners had 80% reciprocity rates (*M* = 5.08, *SEM* = 0.15) than 20% reciprocity rates (*M* = 4.63, *SEM* = 0.18)(*p* = 0.01, *Cohen’s d* = 0.22). This effect was not found for participants in the social Task Version (80% reciprocity rate: (*M* = 4.89, *SEM* = 0.16), 20% reciprocity rate: (*M* = 4.90, *SEM* = 0.20)(*p* = 0.99, *Cohen’s d* = − 0.004). Partners that participants indicated that they did not encounter (7 on a 7-point scale) were not presented in later trials of post-experiment ratings.

**Fig. 9:**
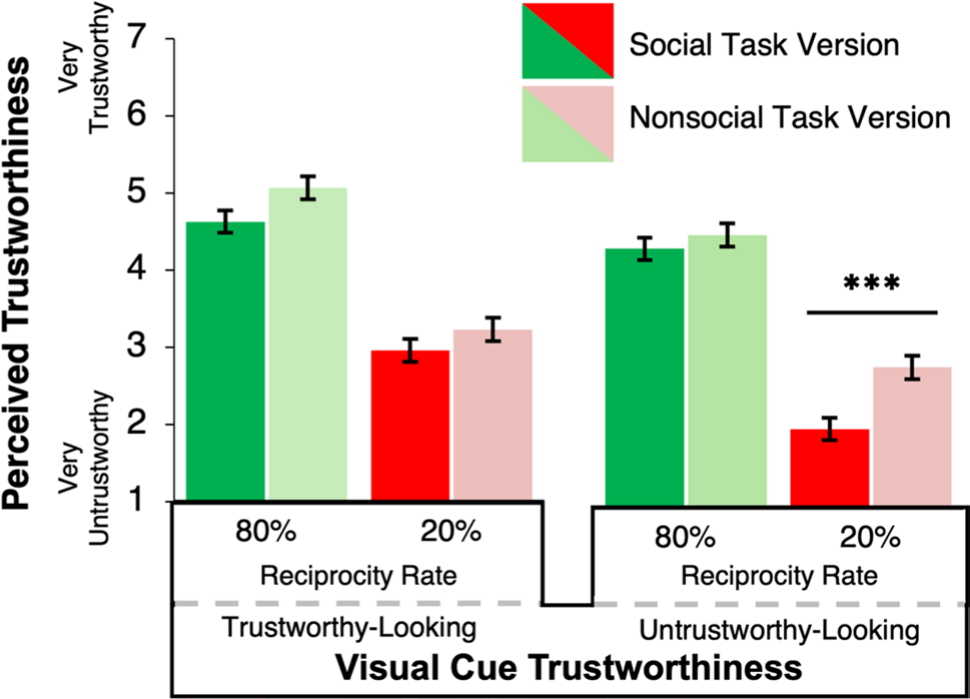
Perceived trustworthiness ratings for encountered partners by Visual Cue Valence, Reciprocity Rate, and Task Version. *Note.* Error bars represent ± SEM. A significant Reciprocity Rate × Visual Cue Trustworthiness × Task Version interaction was observed for perceived trustworthiness ratings of encountered partners. In the social Task Version, Visual Cue Trustworthiness influenced perceived trustworthiness only when partners displayed untrustworthy behaviour (20% Reciprocity Rate). In the nonsocial Task Version, Visual Cue Trustworthiness did not significantly affect ratings, although the trend was in the expected direction. Pairwise comparisons by Reciprocity Rate revealed a significant effect of Visual Cue Trustworthiness only in the social Task Version at the 20% reciprocity level (*P < .*001). Multiple comparisons were adjusted using the Least Significant Difference (LSD) method. *** *P < *.001.

**Fig. 10:**
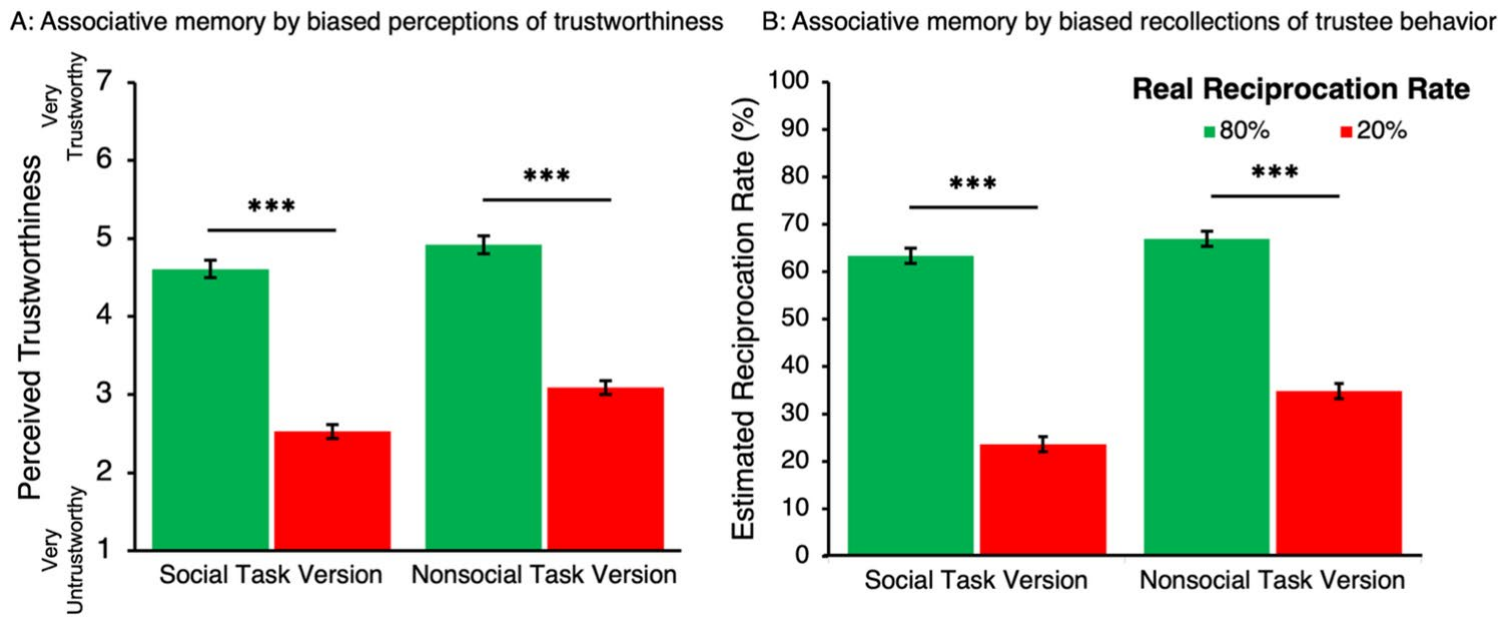
Associative memory for trustee counterparts in Experiment 2. Panel A: Average perceived trustworthiness ratings by Task Version (social vs. nonsocial) and Reciprocity and Reciprocity Rate. Panel B: Estimated reciprocation rates by Task Version and Reciprocity Rate, used as a behavioral measure of associative memory. A significant Reciprocity Rate × Task Version interaction was observed only for estimated reciprocation rates (Panel B), indicating that participants retained stronger associative memory for partner behavior in the social Task Version than in the nonsocial version. No such interaction was found in perceived trustworthinesss rating (Panel A) despite visual similarity in bar patterns. *Note. *Error bars represent ± 1 SEM. *** *P* < .001.

Ratings of perceived trustworthiness were collected for each of the four partners the participant encountered during the game. A 2 (Task Version: Social, nonsocial) × 2 (Reciprocity Rate: High reciprocity rate, low reciprocity rate) × 2 (Visual Cue Trustworthiness: Trustworthy-looking, untrustworthy-looking) mixed factor ANOVA with betrayal trauma added as a control factor was conducted on ratings of perceived trustworthiness for encountered partners. Consistent with *Hypothesis 3a*, a significant main effect of Visual Cue Trustworthiness was observed on perceptions of trustworthiness for encountered partners (*F*(1,193) = 5.99, *p* = 0.02, *η*^2^ = 0.01), where partners with trustworthy-looking visual cues (*M* = 3.97, *SEM* = 0.08) were perceived as more trustworthy post-task than partners with untrustworthy-looking visual cues (*M* = 3.60, *SEM* = 0.07). This main effect of Visual Cue Trustworthiness was not modulated by Task Version, indicating a lack of support for *Hypothesis 3b (p* = 0.45, *η*^2^ < 0.001). Furthermore, as shown in Fig. 9, a significant Reciprocity Rate by Visual Cue Trustworthiness by Task Version interaction was found (*F* (1,193) = 4.74, *p* = 0.03, *η*^2^ = 0.004), which seemed to suggest that in the social Task Version, partners did not reward trustworthy visual cues in their investment behavior while behavior (Reciprocity Rate) is high/trustworthy. However, perceptions of trustworthiness are largely impacted by untrustworthy behavior, especially when paired with untrustworthy-looking visual cues, in which case the visual cues do impact ratings of perceived trustworthiness. In the nonsocial Task Version, participants seem to use both visual and behavioral information when reporting impressions of perceived trustworthiness (see Fig. 9). Results show a significant main effect of Reciprocity Rate (*F* (1,193) = 157.41, *p* < 0.001, *η*^2^ = 0.18) where partners with high reciprocity rates (*M* = 4.76, *SEM* = 0.08) were rated to be perceived as more trustworthy than partners with low reciprocity rates (*M* = 2.81, *SEM* = 0.08) (see Fig. 10a). Unlike Experiment 1, no significant interaction was found between social Task Version and Reciprocity Rate (*F* (1,193) = 1.09, *p* = 0.30, *η*^2^ = 0.001). This finding suggests that partners’ behavior during the Trust Game was similarly influential for both social and nonsocial Task Versions when making post-experiment perceptions of trustworthiness. Lastly, a significant main effect of Task Version was found (*F*(1,193) = 15.50, *p* < 0.001, *η*^2^ = 0.02) where participants in the nonsocial Task Version reported higher perceived trustworthiness (*M* = 4.00, *SEM* = 0.08) than participants in the social Task Version (*M* = 3.57, *SEM* = 0.08). No other main effects or interactions in the full factorial ANOVA were found to reach significance (*p* > 0.29).

For each *encountered* partner, participants were asked to estimate the portion of exchanges that the partner chose to reciprocate. Though unknown to the participants, the true rates of reciprocity were 80% and 20%, where y-axis values in Fig. 10b represent the estimated percentage of trials in which reciprocation occurred. A 2 (Task Version: Social, nonsocial) × 2 (Reciprocity Rate: High reciprocity rate, low reciprocity rate) × 2 (Visual Cue Trustworthiness: Trustworthy-looking, untrustworthy-looking) mixed factor ANOVA was conducted on estimated reciprocation rates finding a significant effect of Visual Cue Trustworthiness (*F*(1,164) = 7.01, *p* = 0.01, *η*^2^ = 0.01) where partners with trustworthy-looking visual cues estimated partners’ reciprocation rates to be higher (*M* = 49.72%, *SEM* = 1.15%) than partners with untrustworthy-looking visual cues (*M* = 44.67%, *SEM* = 1.16%), supporting *Hypothesis 3a*. This effect did not interact with Task Version (*p* = 0.69), failing to find support for *Hypothesis 3b*. A significant main effect of Reciprocity Rate was found (*F*(1,164) = 248.87, *p* < 0.001, *η*^2^ = 0.29), where participants estimated a higher reciprocity rate for partners with high reciprocity rates (*M* = 65.13%, *SEM* = 1.21%) than partners with low reciprocity rates (*M* = 29.25%, *SEM* = 1.14%). The interaction between Reciprocity Rate and Task Version was also significant (*F*(1,164) = 5.14, *p* = 0.03, *η*^2^ = 0.01), suggesting that estimations of the portion of reciprocation trials differed between the social and nonsocial Task Versions (see Fig. 10b) complemented by findings using perceived trustworthiness as a measure of associative memory. No other effects in this analysis reached significance (*p* > 0.11).

### Manipulation check

**Fig. 11:**
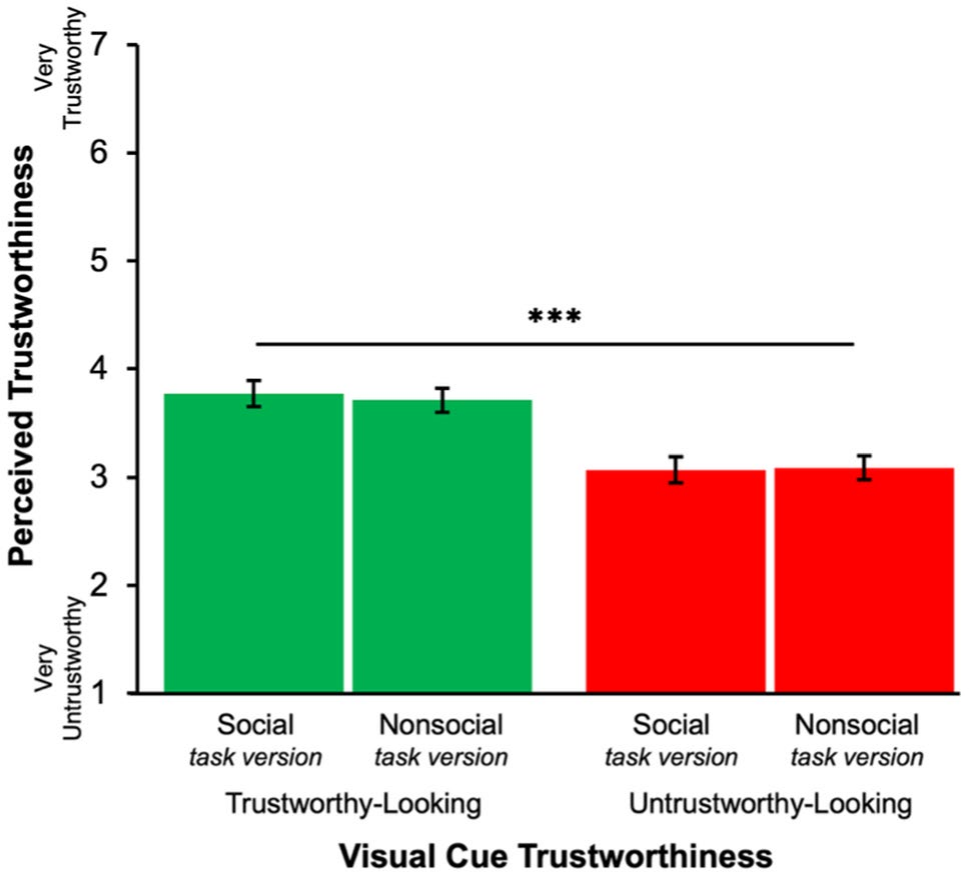
Ratings of Perceived Trustworthiness for unencountered Partners *Note.* Error bars represent ± SEM. Visual cues in the trustworthy-looking condition were rated significantly higher in perceived trustworthiness than those in the untrustworthy-looking condition across both task versions (*P < *.001). The significance bar reflects this main effect of Visual Cue Trustworthiness, confirming the success of the manipulation. Scores reflect ratings of unencountered partners (foils) collected post-task as a manipulation check for the effect of Visual Cue Trustworthiness. *** *P < *.001.

To assess manipulation effects on ratings of perceived trustworthiness by Task Version, we conducted a 2 (Task Version: Social, nonsocial) × 2 (Visual Cue Trustworthiness: Trustworthy-looking, untrustworthy-looking) repeated-measures ANOVA on ratings of perceived trustworthiness for unencountered partners with betrayal trauma added as a control factor. Results showed similar ratings when comparing the social and nonsocial Task Versions, consistent with the results of pilot testing on the new nonsocial stimuli and results of the social Task Version from Experiment 1. Specifically, the results of the ANOVA showed a significant main effect of Visual Cue Trustworthiness (*F*(1,193) = 25.96, *p* < 0.001, *η*^2^ = 0.08; Fig. 11) but no indication of a cue trustworthiness by Task Version interaction (*F*(1,193) = 0.19, *p* = 0.67, *η*^2^ < 0.001) whereas in Experiment 1 this latter trend was marginal. Furthermore, there was no between-subjects effect of Task Version for these ratings of perceived trustworthiness (*p* = 0.87), suggesting that participants did not perceive significantly different impressions of trustworthiness between social and nonsocial counterparts. Thus, the manipulation check confirmed that significant differences in ratings of perceived trustworthiness based on cue trustworthiness did not differ across the social and nonsocial Task Versions.

### Post hoc analysis of believability ratings

To assess whether effects in Experiments 1 and 2 were influenced by the degree of participants’ belief in the cover story (i.e., that they were interacting with real partners), we conducted a post hoc analysis within the social Task Version. Participants were split into high (ratings of 1–3) and low (ratings of 4–6) believability groups based on their responses to the manipulation check item. This analysis was limited to the social Task Version, as believability ratings were not collected in the nonsocial Task Version. Believability Group was included as a between-subjects factor in all primary hypotheses-testing analyses. Results indicated that believability level did not meaningfully alter any reported effects; p-values remained consistent and did not cross the conventional threshold for significance. Thus, the effects observed in the social Task Version were robust across varying levels of belief in the manipulation.

## General discussion

The present study aimed to determine the social relevance of visual cues of trustworthiness to trust-related learning and memory. Past research has consistently supported that static facial characteristics bias perceived trustworthiness and trust behavior. However, despite using nonsocial counterparts as control conditions, past literature has not considered whether the bias observed for faces can be equally observed in nonsocial cues. To address this gap, the present study leveraged the Multi-Round Trust Game and manipulated the trustworthiness of the visual cues representing social and nonsocial partners in social and nonsocial contexts, respectively. We found that visual cues of trustworthiness biased perceived trustworthiness and trust behavior for both social and nonsocial partners. However, differences in results observed across Experiment 1 and 2 indicated that the effects of visual cues of trustworthiness on behavior depended on the salience of the visual cues. Specifically, Experiment 1 indicated that subtle nonsocial visual cues (e.g., red versus green color background) biased perceived trustworthiness but had no bearing on trust-related behavior. However, findings from Experiment 2 suggested that once social and nonsocial visual cues were salient in a trust-related decision context, their impact on behavior was similar to that of facial visual cues found in social contexts. Furthermore, across Experiment 1 and 2, visual cues of trustworthiness continued to bias post-task associative memory for social and nonsocial partners. The implications of these findings for this area of study include theoretical implications that are discussed below.

### Perceived trustworthiness

Available evidence indicates that, in some situations, trust in non-human entities (“nonsocial trustees”) is greater than trust in fellow human entities^13^, though the relationship is complex^61^. Additionally, similar to facial indicators of trustworthiness, nonsocial visual cues can prompt trust behaviors when the meaning of these cues is either implicit^14^ or explicitly provided^16^, resulting in either positively or negatively biased trust behavior^11^. Upon making first impressions, visual cues are used to bias how a counterpart is perceived as trustworthy or untrustworthy. We found a consistent effect of visual cue trustworthiness on ratings of perceived trustworthiness for unencountered partners across the social and nonsocial versions of the task, aligning with our manipulation check results regarding the validity of the visual cues. These findings replicated those of past research on the effect of static facial characteristics on trust perceptions^7,8^, while extending research on impressions of trustworthiness for nonsocial stimuli^14^.

### Initial trust behavior

This study also examined effects of visual cues of trustworthiness on trust behavior across social and nonsocial cue types, to examine whether these appearance-based factors that impact perceptions of trustworthiness also influence behavior. Past literature has found that facial cues of trustworthiness are used to make initial trust decisions^43,44,62^, attributing the effect to “intuitive accessibility”^12^. Although existing literature has not conducted a direct comparison using nonsocial trustees, evidence on the enhanced processing of faces versus non-face stimuli suggests that the intuitive accessibility of nonsocial visual stimuli may be less than that of face stimuli^63^.

Our study found that, in Experiment 1, where nonsocial visual cues were subtle, the effects of Visual Cue Trustworthiness on initial trust-related behavior were stronger in the social than in the nonsocial Task Version. However, in Experiment 2, the trustworthiness of the nonsocial visual cues was more salient; we observed an overall effect of visual cue trustworthiness equivalent across social and nonsocial task versions. These findings align with the Betrayal Aversion theory^28^, as participants exhibited more consistent differences in their initial decision to trust social versus nonsocial partners. Only when the visual cues of trustworthiness for nonsocial partners were made more salient (Experiment 2) did we observe an overall effect of visual cue trustworthiness across social and nonsocial contexts. These findings partially supported *Hypothesis 1*, which predicted that social and nonsocial visual cues would have a similar effect on initial trust behavior, highlighting the possible role of cue salience in moderating these effects. This caveat aligns with findings by Pfabigan^22^, which suggest that social stimuli are perceived as comparatively more salient in trust-related contexts.

Initial trust behavior appears to be guided by intuitive, affectively laden judgments formed rapidly during first impressions. *Affect Heuristic Theory*^31^ helps explain why participants may favor visual cues that “feel” trustworthy, particularly in social contexts. Although both social and nonsocial cues provide affective information, there is likely to be a stronger immediate impact of social than nonsocial cues, due to the relatively greater intuitive accessibility and evaluability of social information (i.e., faces). Human perceivers have dedicated neural networks and extensive experience interpreting facial information, but relatively limited frameworks for interpreting visual trust signals from inanimate sources. As posited by the *Evaluability Hypothesis*^32^, more evaluable cues are weighted more heavily in decision making, explaining why subtle facial features influenced trust behavior in Experiment 1, whereas color-coded nonsocial cues (Experiment 1) did not; until the latter were made more salient in Experiment 2.

### Trust-related learning

Learning about partner trustworthiness is conducted across repeated interactions^52^. For instance, prior work has indicated enhanced processing of untrustworthy behavior when a partner is perceived as trustworthy based on their visual cues, making them a “wolf in sheep’s clothing”^64^. Specifically, untrustworthy behavior was remembered better when associated with *initially misleading,* trustworthy-looking visual cues than with untrustworthy-looking visual cues, suggesting a protective mechanism for detecting defectors^64^. Based on these findings, we proposed that trustees with trustworthy-looking cues but an incongruent low reciprocity rate (“wolves in sheep’s clothing”) would receive lower investments across trials than trustees with congruent, untrustworthy-looking cues and low reciprocity (*Hypothesis 2a*). Additionally, we expected that this effect would be enhanced in the social Task Version (*Hypothesis 2b*). While existing literature has described analogous trust-related memory for nonsocial agents such as IoT and automation^61,65^, it has not experimentally manipulated the trustworthiness of the visual cues of nonsocial stimuli within economic decision tasks, in particular those where multiple rounds are played with each partner. Based on literature applying Attribution Theory, finding comparatively lower levels of empathy for nonhuman agents^66^, and enhanced processing of social feedback^22^, we may deduce more attributions to partner agency in social Task Versions than nonsocial Task Versions, thus resulting in faster learning when partners are social.

The use of the Multi-Round Trust Game in the present study not only required participants to act on initial impressions but was furthermore used to model trust learning across iterative interactions with each counterpart (“trustee”). We found that, when averaged across trials, visual cue trustworthiness impacted financial investments, such that participants invested more money in partners with trustworthy-looking visual cues. This finding suggests that visual cues were used to form first impressions that lasted across iterative trials, despite behavioral feedback, which may have been incongruent (i.e., 20% reciprocity rate). However, we did not find support for *Hypothesis 2a* that trustees with trustworthy-looking cues and an incongruent low reciprocity rate would receive lower investments across trials than trustees with congruent, untrustworthy-looking cues and low reciprocity rate. Nor did we find support for *Hypothesis 2b* that this effect was enhanced in the social Task Version. Thus, our findings for average investments somewhat conflict with prior reports of how a wolf in sheep’s clothing affects trust observed in associative memory^64^.

Additionally, over iterative interactions, we found that participants progressively invested less money in their counterparts (i.e., trust decay^67^), though this may be attributed to the fact that all conditions required some degree of betrayal (i.e., no partner reciprocated 100% of the time). These findings align with past research on trust decay in competitive environments^68^, which is notable considering that the Multi-Round Trust Game presents a competitive dynamic between the participant and their counterpart. Although initial investments showed higher investments for trustworthy-looking partners, the differentiation by visual cue trustworthiness was no longer present in the latter half of the 15 trials, as indicated by a significant interaction between Block and Visual Cue Trustworthiness. Nevertheless, participants did invest more in their trustworthy-acting counterparts (80% reciprocation rate) than in their untrustworthy-acting counterparts (20% reciprocation rate).

Participants made significant changes in investment behavior during early trials of the Trust Game, both with social and nonsocial partners, although this was less pronounced for social partners with high reciprocity rates. This finding suggested that although betrayals by social and nonsocial agents are incorporated into trust behavior, positive interactions influence subsequent trust behavior for nonsocial partners more than social partners. Lastly, a three-way interaction of Task Version, Block, and Reciprocity Rate suggests that although untrustworthy-acting partners were punished for their betrayals in both social and nonsocial Task Versions, only participants interacting with nonsocial partners increased their investments over trials when partners acted trustworthily.

### Associative memory

Importantly, effective memory of partner trustworthiness relies upon successful memory of partners’ past actions. Information relating to expectancy violations^35^ and losses^36^ are particularly memorable, however findings showing enhanced memory for cheaters are inconsistent^40,41^. Memory-based impressions are likely to be influenced by factors such as visual cues and reputational information across social and nonsocial contexts; however, existing research suggests that social visual cues may have a stronger impact. Indeed, in social contexts, betrayals are associated with greater memory compared to positive social exchanges^38^. Further, faces may be more easily remembered than complex non-face stimuli^42^.

As expected, across our two experiments and Task Versions, participants’ associative memory for partner trustworthiness was biased by the trustworthiness of the visual cue, supporting *Hypothesis 3a*. Interestingly, partners’ visual cues biased memory of partner trustworthiness, even when prompted specifically to estimate their partners’ Reciprocity Rate (behavior). Across measures and experiments, we did not observe significant differences in the effect of visual cues between the social and nonsocial versions of the task, indicating a lack of support for *Hypothesis 3b*. However, Experiment 2 indicated that negatively visual cues of trustworthiness led to relatively lower ratings of perceived trustworthiness for untrustworthy-acting social partners (20% Reciprocity Rate condition). In contrast, visual cues of trustworthiness did not seem to impact perceived trustworthiness ratings for encountered partners in the nonsocial Task Version. This contrasts with past literature on expectancy violations, which suggests that when trustworthy-looking visual cues are paired with untrustworthy-acting partners, associative memory would be more negatively biased^35^.

Suzuki and Suga^64^ found that participants had more accurate associative memory for partners with trustworthy visual cues who exhibited untrustworthy behavior than trustworthy behavior. While the present study found effects on post-task associative memory of both visual cue trustworthiness and Reciprocity Rate to be consistent across social and nonsocial Task Versions, a significant interaction of social Task Version and Reciprocity Rate showed that Reciprocity Rate influenced post-task associative memory for social partners more than nonsocial partners. Lastly, in the social Task Version, ratings of perceived trustworthiness were driven by Visual Cue Trustworthiness only when associated with untrustworthy behavior (20% reciprocity rate). Participants interacting with nonsocial trustees did not use visual cue trustworthiness when making ratings of perceived trustworthiness, though it trends in the expected direction. These findings aligned with theories of Betrayal Aversion, supporting a general predisposition to trust nonsocial agents more than social agents. This was demonstrated both in regard to initial interactions, where social betrayals were more often anticipated (Betrayal Aversion) and reactivity to trustee betrayals seen through memory across blocks in the Trust Game (Attribution Theory). Our findings on the effects of visual cue trustworthiness advance this area of research by demonstrating similar effects of visual cues on trust-related memory for both social and nonsocial agents when visual cues are salient.

Our study found relatively similar findings across Experiments 1 and 2, with only two exceptions. In Experiment 1, manipulations of visual cue trustworthiness for social partners were conveyed through facial features (foreground), whereas visual cues for nonsocial partners were represented by background colors. Results showed that visual cues marginally interacted with Task Version (social, nonsocial) and Visual Cue Trustworthiness (trustworthy-looking, untrustworthy-looking) on initial investments in the Trust Game, such that there was a marginally greater effect of visual cue trustworthiness in the social than the nonsocial Task Version (Experiment 1 only). In Experiment 2, in contrast, no such interaction was found, which we attribute to modifications made to the visual cues. Background context influences perceptions of facial trustworthiness, with negative background content leading to foreground faces being perceived as less trustworthy. Trustworthiness judgments integrate both facial and contextual threat cues, suggesting that such evaluations are not made in isolation^69^. Experiment 2 addressed this limitation by aligning the location of the visual elements used to modulate impressions of trustworthiness (both in the foreground), thereby enabling a clearer comparison of social versus nonsocial visual cues. This methodological change in Experiment 2 was associated with robust (i.e., equivalent to Experiment 1) effects of Visual Cue Trustworthiness in nonsocial cues.

We did observe discrepant findings across Experiment 1 and 2 for trust behavior across trials, measured by investments in the Trust Game. Experiment 2 resulted in a significant effect of Cue Congruence, where partners whose cues were congruent received slightly larger investments than partners whose cues were incongruent, supporting Hypothesis 2a and contradicting the findings in Experiment 1. This discrepancy across experiments may reflect either the shift from background to foreground cues; or the fact that the nonsocial visual cues in Experiment 2 were more overtly valenced, making their meaning more immediately apparent (i.e., easier to evaluate; in line with the *Evaluability Hypothesis*).

Although our behavioral data suggest participants adjusted their behavior in response to perceived partner trustworthiness, it is important to note that all participants were required to complete every round with every partner. This limits the extent to which we can interpret these responses as reflecting active avoidance. While reduced investment, as measured in our study, may indicate lowered trust, it does not capture the full range of behavioral responses available in real-world interactions, such as withdrawal from or rejection of interaction partners. Future work could explore this important dimension more directly using paradigms that include escape or opt-out options, such as the Centipede Game^70^.

In conclusion, findings from the present study showed that visual cues of trustworthiness similarly affected perceived trustworthiness and trust behavior for both social and nonsocial partners. The relationship between Task Version and associative memory was more nuanced once visual cues of trustworthiness were introduced. Specifically, although visual cues similarly elicited trust and distrust behavior, associative memory for trustees was enhanced for social partners, particularly when those partners acted untrustworthy. Appropriate impressions, trust behavior, and memory can be formed based on visual cues of trustworthiness for both social and nonsocial trustees. However, importantly, difficulties in associative memory suggest a vulnerability post-interaction, specifically for nonsocial trustees. Such findings are important to consider as it becomes increasingly common to interact with nonhuman agents that may have varying degrees of humanness, as well as more generalized cues that elicit the sensation of social interaction.

## Supplementary Information

Below is the link to the electronic supplementary material.

Supplementary Material 1

## Data Availability

The datasets generated during and/or analysed during the current study are available from the corresponding author on reasonable request.
