# Supplementary material for: Social versus nonsocial visual cues of trustworthiness uniquely influence trust related behavior and memory
Source: Sci Rep. 2025 Oct 16;15:36153. doi: 10.1038/s41598-025-17094-y (PMC12533057; doi:10.1038/s41598-025-17094-y)

Supplementary Analysis:

Experiment 1:

We conducted an exploratory analysis to more directly examine effects of cue-outcome congruence/incongruence. This analysis classified trustees with congruent cues (Visual Cue Trustworthiness matched reciprocation tendency) and incongruent cues (Visual Cue Trustworthiness did not match reciprocation tendency). Cue Congruence was computed such that investments made in partners with 20% reciprocity rate were subtracted from $10, resulting in investment behavior that indexes memory more broadly. In this full-factorial 2 (Task Version: social, nonsocial) × 2 (Cue Congruence: congruent, incongruent) × 5 (Block: 1-5), the main effect of Cue Congruence did not significantly affect overall investment in the trust game (*p* = .50, *η*^2^ < .001); further demonstrating a lack of support for *Hypothesis 2a*. The interaction between Cue Congruence and Task Version was not significant (p = .20, *η*^2^ = .001). All other main effects and interactions were also not significant (*ps* > .14).

Experiment 2:

A full factorial 2 (Task Version: social, nonsocial) × 2 (Reciprocity Rate: high, low) × 2 (Visual Cue Trustworthiness: trustworthy-looking, untrustworthy-looking) × 5 (Block: Block 1, Block 2, Block 3, Block 4, Block 5) mixed factor ANOVA was conducted on average investment, with betrayal trauma added as a control factor. We observed a marginally significant interaction between Reciprocity Rate and Visual Cue Trustworthiness (*F*(1,193) = 3.46, *p* = .07, *η*^2^ < .001), which did not further interact with Task Version (*p* = .65, *η*^2^ < .001). This marginally significant effect suggested a trend in the high Reciprocity Rate condition (*F*(1,193) = 5.27, *p* = .02, *Cohen’s d* = .16; a difference not found when Reciprocity Rate was low (*F*(1,193) = .50, *p* = .48, *Cohen’s d* = .04; Figure S1).


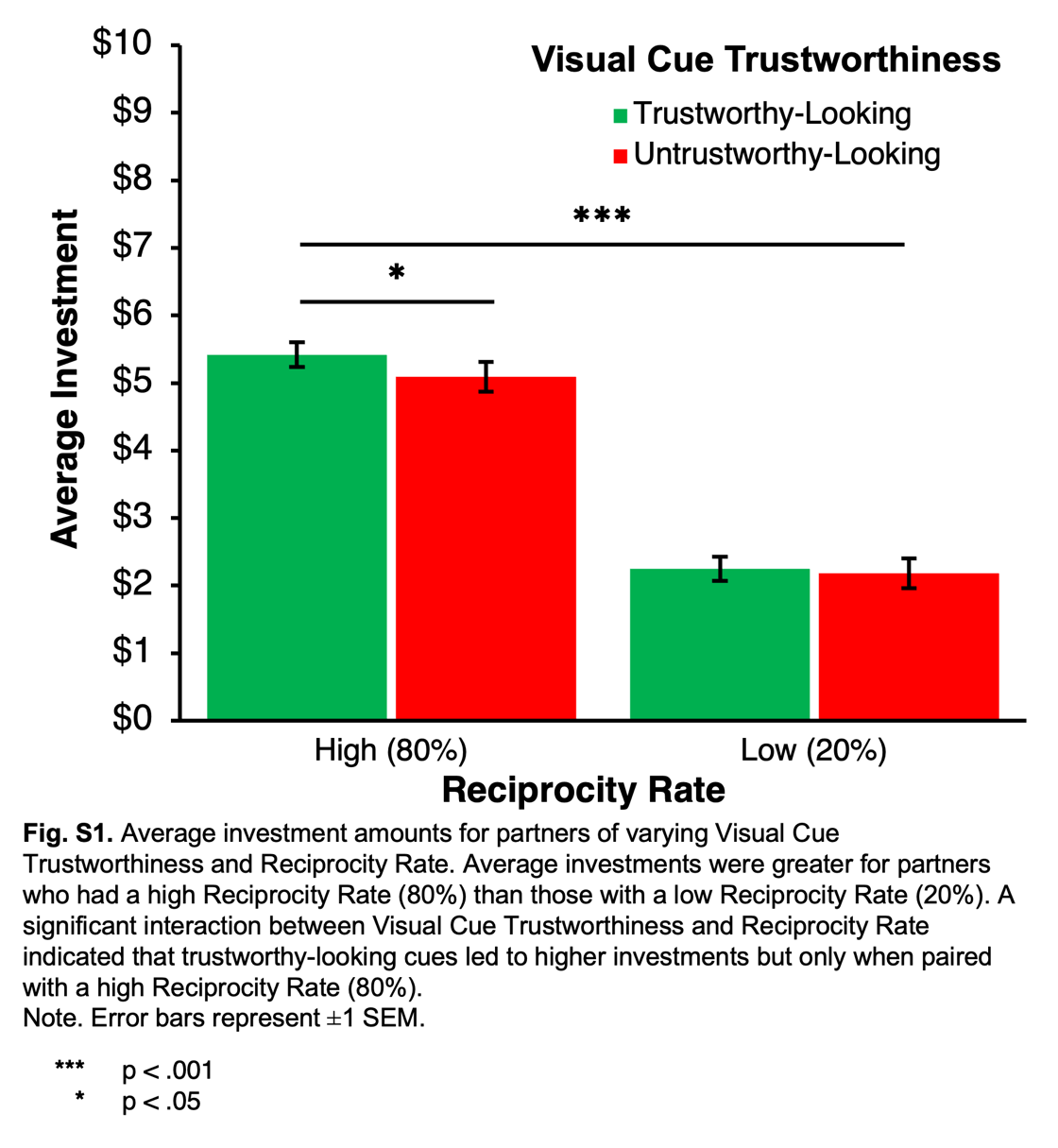


A full factorial 2 (Task Version: social, nonsocial) × 2 (Reciprocity Rate: high, low) × 2 (Visual Cue Trustworthiness: trustworthy-looking, untrustworthy-looking) × 5 (Block: Block 1, Block 2, Block 3, Block 4, Block 5) mixed factor ANOVA was conducted on average investment, again with betrayal trauma added as a control factor. We observed a marginally significant interaction between Block and Visual Cue Trustworthiness (*F*(1,193), *p* = .06, *η*^2^ = .001), where the decline in average investment over blocks was more pronounced in interactions with partners with trustworthy-looking cues. Pairwise comparisons showed a significant difference between Block 1 and Block 5 average investments for partners with trustworthy-looking (*p* < .001, *Cohen’s d* = .37) but not for partners with untrustworthy-looking (*p* = .13, *Cohen’s d* = .18) cues. A significant Block by Reciprocity Rate interaction (*F*(1,193) = 24.31, *p* < .001, *η*^2^ = .01) furthermore suggested increased average investments across blocks for partners with high (80%) but decreased average investments for partners with low (20%) reciprocity rate.

An additional exploratory analysis was conducted, which classified trustees as those with congruent cues (Visual Cue Trustworthiness matches reciprocation tendency) and incongruent cues (Visual Cue Trustworthiness does not match reciprocation tendency). In this full-factorial 2 (Task Version: social, nonsocial) × 2 (Cue Congruence: congruent, incongruent) × 5 (Block: Block 1, Block 2, Block 3, Block 4, Block 5) mixed factor ANOVA again with betrayal trauma added as a control factor, Experiment 2 found a significant effect of Cue Congruence; where partners whose cues (visual and behavioral) were congruent (*M* = 6.62, *SEM* = .08) received slightly larger investments than partners whose cues were incongruent (*M* = 6.42, *SEM* = .08, *F*(1,193) = 9.31, *p* = .003). These findings were generally in support of *Hypothesis 2a* and contrary to findings in Experiment 1. Cue Congruence did not interact with Task Version or Block in this analysis (*p* > .14), thus not supporting *Hypothesis 2b*. No other effects in this analysis reached significance (*p* > .47).

Trust Game Data Collection Script (social task version) (used in Experiment 2 only) (verbally-administered)

Prior to the session's start, Experimenter 1 and Experimenter 2 had the consent form presented on one computer monitor, and the “establishing connection” presentation was played on the neighboring computer monitor.

Experimenter 1 meets the participant at a designated meeting location away from the testing room.

Experimenter 1

Are you here to participate in the trust experiment?

Participant

Yes

Experimenter 1

Ok great! We can go through here to meet another researcher who is currently helping set up with the other participants you’ll be interacting with.

Experimenter 1 walks participant to testing room. Experimenter 1 looks into clipboard as they approach the lab and then up to the sign on the door as they pause to assess which room they should go to.

Experimenter 1

Okay, yeah, so you’re investor 1….let’s see… you’re going to be in room 303H. The other participants you will be playing with are currently being set up in other rooms around the building.

Experimenter 1 asks the participant to turn off electronic devices and place it on the table behind them. Do not persist if they resist.

Experimenter 1 reminds the participant that the task will take up to 30 minutes and to use the restroom if they would like.

Experimenter 1 asks the participant to state their SONA identity code aloud in order to verify their identity with our records.

Experimenter 1

The purpose of the study is to investigate how trust is built and maintained among adults, and how that affects decision-making. This form describes your rights as a participant. Please read it carefully and let me know if you have any questions. If you consent to participate, you will select “yes”. Let me know if you would like a copy of this form. This form also states that you will be paid for your performance in the study. This means that you will earn more money in real life depending on how much money you earn in the task, up to $4 in an Amazon Gift Card.

Experimenter 1 turns to Experimenter 2 and addresses them casually but impatiently.

Experimenter 1

Ugh in 203 it says they’re still not ready. Can you go and see if they’re ready?

Experimenter 2

Ok, sure

Experimenter 2 leaves the room, closes the door, and waits for 2 minutes to pass.

Experimenter 1

We can continue as soon as s/he gets back - she is checking to make sure the other participants you'll be interacting with are ready to begin.

Experimenter 1 notices that the participant has agreed to the consent form and has progressed to the demographics portion of the procedure.

Experimenter 1

Ok now that you’ve consented to participate I can set you to “ready” on my end.

Experimenter 1 theatrically interacts with the system to switch participant status to “ready”.

…some time passes; Experimenter 1 notices that the participant has completed the demographics portion of the procedure, and that the Trust Game is loading.

Experimenter 1

Ok just wait right there until she gets back. We just need to make sure everyone is ready before we begin. It still looks like on my end that we’re still waiting on someone.

Experimenter 1 uses the concealed computer mouse to advance the system display to show all players as “ready”.

Experimenter 1 theatrically notices that the system shows that everyone is ready.

Experimenter 1

Ok! Everyone’s ready! You can click the link to begin the experiment. The experimental procedure will take up to 30 minutes. Do you have any questions before we start?

Participant

No

The participant clicks “ok” and begins the Trust Game instructions on their computer.

After 2 minutes has passed since Experimenter 2 left the room, Experimenter 2 returns

Experimenter 2

The participants in the other rooms are ready. We can start. They were just running a little behind but all’s good.

Experimenter 1

Thank you! I saw it pop up on here while you were gone! I appreciate it anyway!

The experimental procedure commences. Experimenters sit quietly in the room with the participant for the remaining duration of the session until the participant is notified on screen that they have completed the procedure.

[End of verbally-administered instructions / cover story]

On-screen instructions digitally-administered prior to the Trust Game in Experiment 2. Experiment 1 had very minor version-specific differences.

1. Welcome to the Learning to Trust Study!
2. This study investigates how trust is built and maintained among adults, and how that affects decision-making.

We will first go over instructions and a few practice rounds.

1. (*social A*) In your role, you will not have a photo to represent you. Your partners will choose a photo that represents them from a photoset.
2. (*nonsocial A*) The computer programs that you play with will be represented by computer avatars with different wallpaper backgrounds.
3. (*social B*) You will be interacting with 4 different partners who were also scheduled at this time.

After you finish the round with each partner, you will be randomly matched with a new partner.

1. (*nonsocial B*) You will be interacting with 4 different computer programs.

After you finish the round with each computer, you will be randomly matched with a new computer program.

1. (*social C*) You will play a game that involves 2 players. You will be one of those players.

You will be assigned a role, and you will play in that role for the entire experiment.

1. (*nonsocial C*) You will play a game that involves 2 players. You will be one of those players. The other player will be a computer program.
2. In the game, you’ll be given a $10 endowment for each investment. You will then decide how much of the $10 you would like to invest in your partner. Any amount between $0 and $10 can be invested.
3. Any money invested in quadrupled by the time it reaches your partner. For example, if you invest $5, your partner will receive $20. Any money not invested is simply kept by you.

You will be assigned a role, and you will play in that role for the entire experiment.

1. Each time that an investment is made, your partner can either...

a) keep all of the money, or

b) share by returning 50% to you

1. You will now perform 2 practice exchanges with a computer program.
2. Use your mouse to indicate how much you would like to invest. Click on the number you would like to invest. You can invest any amount from $0 to $10.
3. (*social D*) In later trials, you will see a photo your partner has picked to represent themselves. In this practice round, you will have 2 exchanges with the same computer program. In each round, you will be given $10 and you will decide the amount you would like to invest in your partner.
4. Remember, you will receive up to a $4 Amazon Gift Card depending on your performance. Try to earn as much money as possible.

***2 practice trials***

1. Great work! You may now begin with the experimental trials.

When you press the spacebar, we will match you with another participant scheduled at this time.

You will interact with 4 partners in total. You will have 15 exchanges with each of your partners.

1. (*social E*) Please wait while we match you with a partner. The session will begin once a partner is found.

This will take no longer than 20 seconds.

*Excerpt from digitally-administered consent form describing compensation:*

…

If you agree to take part in this research study, you will receive SONA credits for completion of the task *and* up to $4 in the form of an Amazon Gift Card, dependent on your performance in the task (20% of the payout on a randomly selected trial). A valid email address will be collected to distribute the gift card. Upon completion, you will be awarded SONA credits which may be used as extra/research credit in previously approved psychology courses. If you choose not to participate, you may notify your instructor and ask for an alternative assignment of equal effort for equal credit. There will be no penalty.

…

*Phrasing of digitally-administered question assessing degree of belief in the social manipulation (social condition only):*

“As you read before, we’re interested in how people make decisions in different economic situations. We have been running this study for a while and have received feedback from past participants. Some subjects have wondered if these people are in fact real. They are indeed real.

However, it is important for us to know whether you doubted whether the feedback you were seeing was real or not.

On a scale from 1-7, where 1 represents ‘completely believed’ and 7 represents ‘didn’t believe at all’, how much did you believe you were playing with real partners?”

Signs hung on testing-room door (used in Experiment 2 only)

Social Task Condition


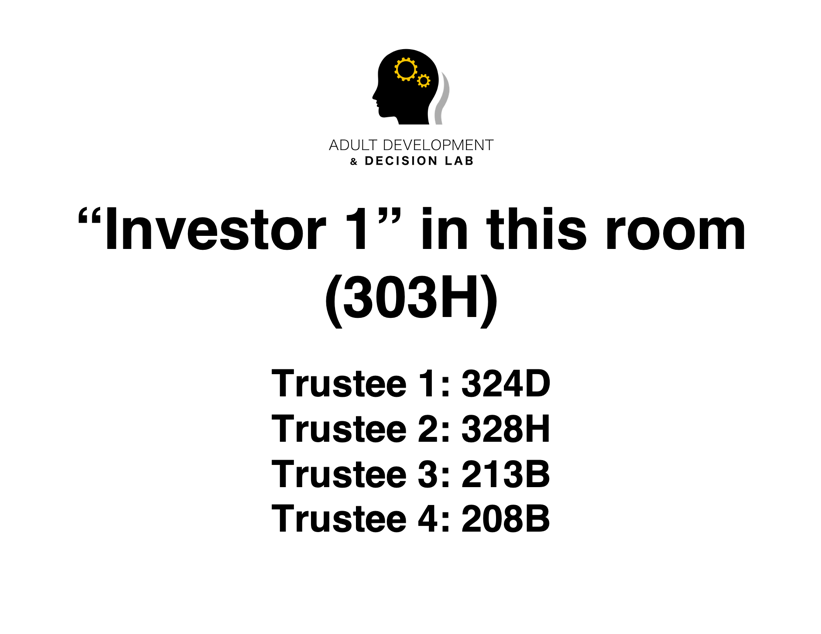


Nonsocial Task Condition


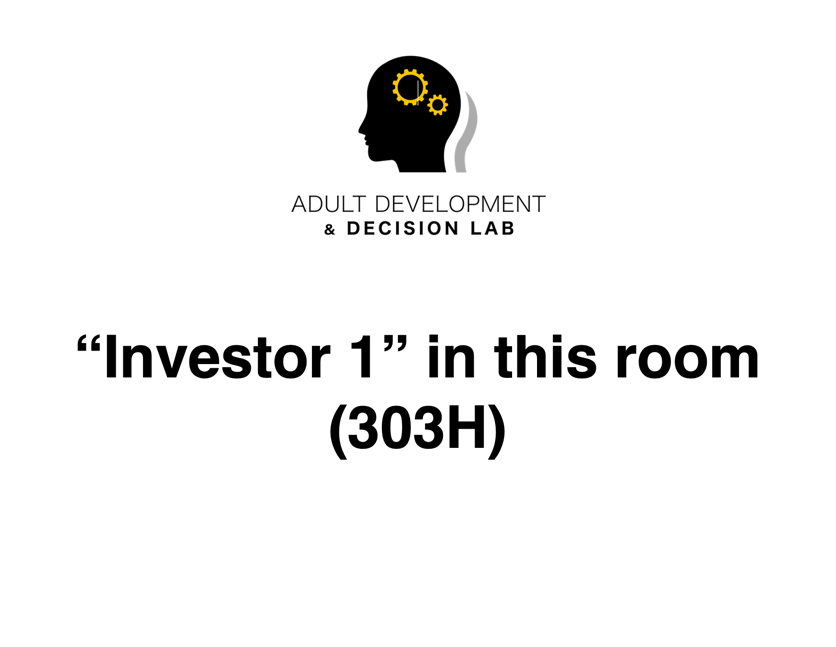

Supplement: Supplementary file 1 — Supplementary Material 1 [file 41598_2025_17094_MOESM1_ESM.docx]
